# Genetic Differentiation of North-East Argentina Populations Based on 30 Binary X Chromosome Markers

**DOI:** 10.3389/fgene.2018.00208

**Published:** 2018-06-13

**Authors:** Gabriela P. Di Santo Meztler, Santiago del Palacio, María E. Esteban, Isaías Armoa, Carina F. Argüelles, Cecilia I. Catanesi

**Affiliations:** ^1^Laboratorio de Diversidad Genética, IMBICE, CICPBA-Consejo Nacional de Investigaciones Científicas y Técnicas, La Plata, Argentina; ^2^Instituto Argentino de Radioastronomía, CCT-La Plata, Consejo Nacional de Investigaciones Científicas y Técnicas, CICPBA, Villa Elisa, Argentina; ^3^Secció de Zoologia i Antropologia Biológica, Facultat de Biologia, Universitat de Barcelona, Barcelona, Spain; ^4^Institut de Recerca de la Biodiversitat, Universitat de Barcelona, Barcelona, Spain; ^5^Dirección de Desarrollo e Integración Regional Municipalidad de Eldorado, Misiones, Argentina; ^6^LACyGH-GIGA, Instituto de Biología Subtropical, nodo Posadas-Dpto. de Genética-FCEQyN UNaM-Consejo Nacional de Investigaciones Científicas y Técnicas, Posadas, Argentina; ^7^Cátedra de Genética, Facultad de Cs. Naturales y Museo, UNLP, La Plata, Argentina

**Keywords:** polymorphism, X chromosome, genetic variation, Alus, INDELs, SNPs, Argentina

## Abstract

Alu insertions, INDELs, and SNPs in the X chromosome can be useful not only for revealing relationships among populations but also for identification purposes. We present data of 10 Alu insertions, 5 INDELs, and 15 SNPs of X-chromosome from three Argentinian north-east cities in order to gain insight into the genetic diversity of the X chromosome within this region of the country. Data from 198 unrelated individuals belonging to Posadas, Corrientes, and Eldorado cities were genotyped for Ya5DP62, Yb8DP49, Ya5DP3, Ya5NBC37, Ya5DP77, Ya5NBC491, Ya5DP4, Ya5DP13, Yb8NBC634, and Yb8NBC102 Alu insertions, for MID193, MID1705, MID3754, MID3756 and MID1540 Indels and for rs6639398, rs5986751, rs5964206, rs9781645, rs2209420, rs1299087, rs318173, rs933315, rs1991961, rs4825889, rs1781116, rs1937193, rs1781104, rs149910, and rs652 SNPs. No deviations from Hardy-Weinberg equilibrium were observed for Posadas and Corrientes. However, Eldorado showed significant values, and it was found to have an internal substructuring with two groups of different origin, one showing higher similarity with European countries, and the other with more similarities to Posadas and Corrientes. *F*_st_ pairwise genetic distances emerged for some markers among the studied populations and also between our data and those from other countries and continents. Of particular interest, Alu insertions demonstrated the most differences, and could be of use in ancestry studies for these populations, while INDELs and SNPs variation were informative for differentiation within the country.

## 1. Introduction

The human X chromosome contains multiple types of non coding markers distributed along its sequence, including Alu insertions, insertion-deletions (INDEL), and single nucleotide polymorphisms (SNP) markers. Alu insertions are short interspersed nuclear elements classified into 12 subfamilies that appeared at different times during primate evolution (Kapitonov and Jurka, 1996). Alu sequences are about 300 bp in length and were ancestrally derived from the 7 SL RNA gene, inserted into the genome through an intermediate RNA single strand generated through transcription by RNA polymerase III (Batzer and Deininger, 1991). These polymorphisms consist of the presence or absence of Alu elements at a particular locus. Usually, they are selectively neutral and, as their location hardly changes or rearranges, they are considered to be derived from one unique event in which the absence of the insertion is the ancestral state for Alu markers (Batzer et al., 1994). All of these distinctive features make the human Alu insertion polymorphisms a good tool for studying the genetic variation and the evolutionary relationships of human populations (Stoneking et al., 1997). INDELs and SNPs have many genetic advantages for population studies: (i) they are widely spread throughout the genome, including the X chromosome; (ii) the majority of these polymorphisms derive from a single mutation event; (iii) mutation rates are much lower than those of repetitive markers; (iv) they can show significant allele frequency differences among geographically distant populations; and (v) they can be easily genotyped, even from degraded DNA samples, given the short length of amplicons (Tomas et al., 2008; Pereira et al., 2009; Ribeiro-Rodrigues et al., 2009; Casto et al., 2010; Li et al., 2010).

Concerning transmission properties, the X chromosome is gaining significant importance in population and forensic genetic studies. The mammalian X chromosome presents many unique features. Females inherit an X chromosome from each parent, whereas males inherit a single, maternal X chromosome. In the female germinal cell line, both X chromosomes undergo recombination, whereas in males, instead, recombination is restricted to the short regions at both tips of the X chromosome arms that recombine with equivalent segments on the Y chromosome. Some interesting characteristics of the X chromosome rely on its special transmission pattern: (i) it travels between both sexes in each generation, telling a story which differs from uniparental genomes; (ii) it has an overall genetic diversity within populations higher than autosomes due to its reduced effective population size, which makes it more sensitive to the effects of genetic drift, population substructuring and selective sweeps; (iii) the lower rate of recombination leads to an increase in levels of linkage disequilibrium (LD) in comparison to autosomes; and (iv) the hemizygous state in males provides a direct access to haplotypes (Hamosh et al., 2000; Schaffner, 2004).

In Argentina, the populations of different provinces came from multiple, distinct origins. In the case of the Argentinian Mesopotamia (the Misiones, Corrientes, and Entre Ríos provinces) the Guaraní people, who came from Amazonia, settled in the current Argentinian territory between the end of the fifteenth century and the beginning of the sixteenth century (Heguy, 2012). They entered in a violent way, thus generating a situation of continual conflict concerning access to resources by the native non-Guaraní populations who inhabited the region concerning access to resources (Vara, 1985). About a 100 years later, the Jesuits arrived during the European invasion of the area and established Jesuitic missions. This halted the expansion of the Portuguese, who were hunting the aboriginal people for. The Guaraní military men also participated in numerous campaigns of punishment against other aboriginal tribes of the Gran Chaco region, such as the Guaykurú, the Payagú, and the Mbyá. As a consequence of the Bourbon reforms, the Jesuits were expelled from America, and natives emigrated to Corrientes and Santa Fe for working as craftsmen or farmers, while others returned to the jungle (González and Pérez, 2000; Heguy, 2012).

On the other hand, European immigration in Misiones was characterized by two types of settlements: one official (1883–1927) and one private (1920–1945). Private colonization took place in Eldorado, Puerto Rico and Montecarlo, in the northern region of Misiones, where companies of European origin promoted the arrival of foreigners, preferably Germans (Junta de Estudios Históricos del Municipio de Eldorado, 2015; Poenitz, 2015).

Finally, in the case of the Corrientes province, at the end of the fourteenth century this region had substantial ethnic diversity (Caingang, Abipon, Chaná, Caracará, Mocoretá) that changed with the arrival and expansion of the Guaraní people. As a consequence of this expansion, the language and customs of extant communities was replaced by those of the Guaraní. In particular, the city of San Juan de Vera de la Siete Corrientes was founded by Spaniards and their descendants born in America (dubbed “criollos”), who employed these lands as farms for the breeding and exploitation of cows and horses. The initial settlers of the city of Corrientes gradually joined the local Amerindian population (Vara, 1985). In contrast with the situation in Misiones, there were no Jesuitic missions in the province of Corrientes, except for a few in the west coast of the Uruguay River, and the Franciscan mission of Itatí located near the city of Corrientes (Pérez, 1936; Heguy, 2012).

In this work, we studied 10 Alu insertions, 5 INDELs, and 15 SNPs to gain insight into the genetic composition of these particular populations. First, we focused in discerning the different contribution of European and Native American genetic components on the studied populations to understand the diversity that they represent. Second, we analyzed the different information that each kind of marker can offer. This constitutes the first study in South American populations that uses this set of 30 markers.

## 2. Materials and methods

### 2.1. DNA samples

**Figure 1:**
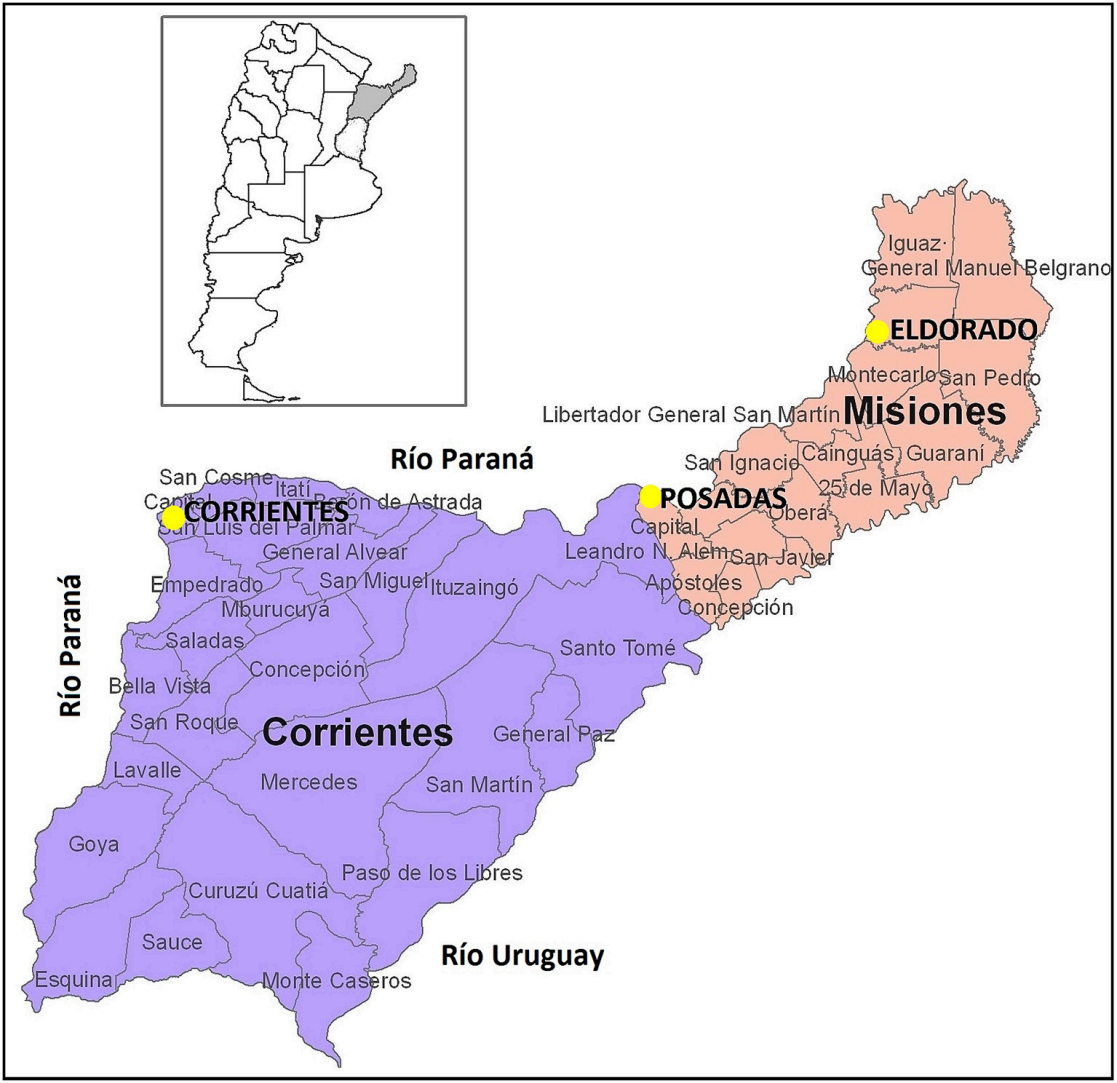
Geographical location of the populations analyzed. Figure adapted from www.cepal.org/.

We analyzed a total of 198 samples from healthy, unrelated persons of both sexes from three different locations in Argentina: the capital city of Corrientes (Corrientes, *n* = 92; 32 females, 60 males), the capital city of Misiones (Posadas; *n* = 52, 28 females, 24 males), and another important city of Misiones (Eldorado, *n* = 54); the geographical location of these cities is shown in Figure 1. Samples from Posadas were obtained in public hospitals; samples from Eldorado were collected in a private laboratory of clinical analysis, and those from Corrientes were collected during a campaign by physician Darío Martín González. The current geographical location and the geographical origin of parents and grandparents of each donor were considered. Concerning Eldorado, samples were separated into two groups according to the grandparents reported origin. We labeled as Eldorado A (*n* = 27; 13 females, 14 males) the donors who knew that their four grandparents were German and/or Swiss, and we labeled donors who did not know the origin of all four grandparents as Eldorado B (*n* = 27; 11 females, 16 males). All samples were obtained with informed consent and were analyzed anonymously; the project was approved by the Ethics Commitee at IMBICE. DNA was isolated from buccal cells and peripheral blood samples as in Gemmell and Akiyama (1996).

### 2.2. Genetic determinations

All PCR amplifications were performed in 10 ul reactions. The Alu sequences analyzed were Ya5DP62, Yb8DP49, Ya5DP3, Ya5NBC37, Ya5DP77, Ya5NBC491, Ya5DP4, Ya5DP13, Yb8NBC634, and Yb8NBC102, and primer sequences were obtained from previous works, as well as amplification and electrophoresis conditions with some modifications (Callinan et al., 2003) (Tables S1, S2). In the case of the 5 INDELs studied, MID193, MID1705, MID3754, MID3756, and MID1540, primer sequences, amplification and electrophoresis conditions are detailed elsewhere (Freitas et al., 2010; Córdoba et al., 2012). Lastly, the 15 SNPs analyzed were rs6639398, rs5986751, rs5964206, rs9781645, rs2209420, rs1299087, rs318173, rs933315, rs1991961, rs4825889, rs1781116, rs1937193, rs1781104, rs149910, and rs652; primer sequences, amplification and electrophoresis conditions were developed in our lab (Tables S3, S4). The location of all markers is shown in Table 1. Genotypes were identified by agarose electrophoresis of the PCR products, followed by Gel Red™ (Biotium) staining and observation under UV fluorescence (Figures S1, S2). Positive and negative controls were used in all the PCR runs to assess the reliability of the determinations.

**Table 1:** Location in the X-Chromosome of the analyzed polymorphisms.

**Marker**	**Position**
Ya5DP3	4,095,243-4,260,035
Ya5DP4	4,670,075-4,850,396
rs6639398	5,223,258
Ya5 491	9,810,906-9,875,672
Ya5DP13	21,230,949-21,438,905
rs5986751	25,199,129
rs5964206	42,739,471
rs9781645	53,765,102
rs2209420	69,328,318
rs1299087	83,336,462
Yb8NBC634	92,693,201-92,890,811
rs318173	97,826,213
rs933315	107,355,477
Yb8NBC102	114,555,491-114,677,890
Ya5DP62	114,555,491-114,677,890
MID 3754	117,768,025
MID 3756	117,768,148
Ya5NBC37	120,184,952-120,332,053
rs1991961	120,692,753
rs4825889	124,185,915
rs1781116	124,186,392
rs1937193	124,187,206
rs1781104	124,187,767
rs149910	127,191,155
rs652	128,147,419
MID1705	128,307,583
Yb8DP49	129,115,374-129,275,464
Ya5DP77	140,674,109-140,839,680
MID193	142,185,283
MID1540	142,194,021

*The location of the Alu polymorphisms was obtained from Callinan et al. (2003) and the location of the INDEL and SNP polymorphisms were obtained from NCBI (1988)*.

### 2.3. Statistical analysis

Allele frequencies were calculated using the software RStudio v.0.99.893 (R Core Team, 2008). Heterozygosity and Hardy-Weinberg equilibrium (HW) were calculated only for female subsamples, while LD was calculated for male. *F*_st_-values were estimated using both female and male data, employing the program ARLEQUIN v.3.5 (Excoffier and Lischer, 2010). Program Past was used to make MDS graphics (Hammer et al., 2001). To infer the population structure, the program STRUCTURE v.2.1 (Hubisz et al., 2009) was used assuming a model of *k* population groups, with *k* between 1 and 5; all runs were performed using a burn-in period of 10^5^ iterations followed by 10^5^ iterations, and a repetition number of 10. To choose the best *k* in each case, Structure Harvester software was used (Earl and vonHoldt, 2012). Intercontinental comparisons were performed using data from Athanasiadis et al. (2007) and Gayà-vidal et al. (2009), including sub-Saharan Africa (Ivory Coast), North Africa (Moroccan High Atlas, Siwa oasis in Egypt, and Tunisia), Greece (Crete Island), Spain (Basque Country), and Native Americans from Bolivia (Aymará and Quechua speakers from Andean region). The comparisons were made with the mentioned populations because there was no data available for other communities of Europeans or Native Americans in the scientific literature. Finally, the calculations of the forensic parameters *PIC* (polymorphic information content), *PE* (power of exclution) and *PD* (power of discrimination) were calculated with an online software ChrX-STR.org 2.0 Calculator (http://www.chrx-str.org/) (Szibor et al., 2006).

## 3. Results

The DNA data corresponding to this work are deposited in http://hdl.handle.net/10915/66905

Genotype frequencies are included as supplemental material (Tables S5–S7). All four populations adjusted to HW equilibrium for most of the polymorphisms analyzed (Tables S8–S11); the exceptions were MID3754 for Posadas and EldoradoB (*p*−values = 0.01), rs1781104 for Posadas (*p*−values = 0.04), Yb8NBC634 for EldoradoA (*p*−values = 0.04), and Yb8NBC102, rs9781645, 1781104 for Corrientes (*p*−value = 0.01, *p*−value = 0.04, *p*−value = 0.03), respectively. Allele frequencies of all populations are shown in Table 2; two of them, Ya5NBC491 and Ya5DP4, were monomorphic for two and three populations, respectively, while Ya5DP13 was monomorphic for all. The average observed heterozygosity (OH) was also analyzed for each population, obtaining values of 0.43, 0.35, 0.40, and 0.41 for Posadas, Corrientes, Eldorado A, and Eldorado B, respectively. Only in the case of Posadas was the observed average heterozygosity slightly higher than the expected one (expected heterozygosity, EH = 0.41), although it was not statistically significant. The *F*_st_-values of the Alu insertions were not significantly different for the populations analyzed. On the other hand, INDELs showed significant *F*_st_-values for most of the comparisons. In the case of SNPs, they showed significant *F*_st_-values for the comparisons between Posadas-Corrientes and EldoradoA-EldoradoB (Table 3).

**Table 2:** Allele frequencies of the 10 Alu sequences, 5 INDELs, and 15 SNPs for the populations analyzed (Insertion +) and global MAF (according to NCBI) for SNPs.

**Marker**	**Marker name**	**Pos**	**Cor**	**Eld A**	**Eld B**
Alu	Ya5DP3	0.17	0.10	0.08	0.11
insertions	Ya5DP4	0	0.02	0	0
	Ya5NBC491	0.99	0.99	1	1
	Ya5DP13	1	1	1	1
	Yb8NBC634	0.98	0.98	0.95	0.97
	Yb8NBC102	0.75	0.71	0.71	0.92
	Ya5DP62	0.75	0.70	0.79	0.92
	Ya5NBC37	0.21	0.25	0.32	0.26
	Yb8DP49	0.86	0.85	0.73	0.83
	Ya5DP77	0.74	0.75	0.85	0.74
INDELs	MID3754	0.60	0.56	0.79	0.50
	MID3756	0.68	0.52	0.32	0.44
	MID1705	0.59	0.81	0.85	0.50
	MID193	0.44	0.52	0.72	0.53
	MID1540	0.57	0.60	0.48	0.47
SNPs	rs6639398	0.16	0.15	0.28	0.26
	rs5986751	0.49	0.62	0.50	0.39
	rs5964206	0.32	0.34	0.03	0.47
	rs9781645	0.40	0.61	0.38	0.68
	rs2209420	0.42	0.32	0.35	0.16
	rs1299087	0.39	0.46	0.43	0.41
	rs318173	0.29	0.14	0.35	0.21
	rs933315	0.49	0.43	0.18	0.47
	rs1991961	0.41	0.35	0.5	0.53
	rs4825889	0.30	0.39	0.36	0.31
	rs1781116	0.70	0.58	0.65	0.65
	rs1937193	0.31	0.42	0.33	0.33
	rs1781104	0.59	0.46	0.40	0.47
	rs149910	0.46	0.39	0.32	0.50
	rs652	0.40	0.28	0.19	0.55

**Table 3:** *F*_st_-values for Alu, INDELs, SNPs, and the 30 markers for all the analyzed populations: Posadas, Corrientes, Eldorado A, and Eldorado B.

**Markers**	**Pos-cor**		**Pos-EldA**		**Pos-EldB**		**Cor-EldA**		**Cor-EldB**		**EldA-EldB**	
	***F*_st_**	***p*-value**	***F*_st_**	***p*-value**	***F*_st_**	***p*-value**	***F*_st_**	***p*-value**	***F*_st_**	***p*-value**	***F*_st_**	***p*-value**
Alu	−0.005	0.72	−0.014	0.90	0.007	0.24	−0.028	0.99	0.015	0.11	0.005	0.38
INDELs	0.024^*^	0.02	0.136^*^	0.00	0.019	0.10	0.067^*^	0.00	0.034^*^	0.01	0.116^*^	0.00
SNPs	0.015	0.05	0.010	0.17	0.003	0.22	0.009	0.21	0.018	0.13	0.051^*^	0.00
Alu-INDELs-SNPs	0.013	0.05	0.044^*^	0.00	0.008	0.13	0.018^*^	0.03	0.022^*^	0.04	0.062^*^	0.00

Values marked with ^*^ (highlighted in bold) correspond to p-values < 0.05. Pos, Posadas; Cor, Corrientes; EldA, EldoradoA; EldB, EldoradoB.

The *F*_st_-values and *p*−values for the Alu insertions were also calculated using other populations from previous works (Athanasiadis et al., 2007; Gayà-vidal et al., 2009) (Table 4).

**Table 4:** Below diagonal: *p*-values and above diagonal *F*_st_ between the populations analyzed and other populations of the world.

	**Cor**	**Pos**	**EldA**	**EldB**	**Iv coast**	**Crete Is**	**Basq C**	**Siwa O**	**M H Atlas**	**Tun**	**Ay sp**	**Q sp**
Cor	^*^	0.53	0.36	0.97	**0.03**	0.29	0.14	0.08	**0.00**	**0.00**	**0.00**	**0.00**
Pos	−0.004	^*^	0.15	0.67	**0.01**	0.08	**0.02**	0.42	**0.00**	**0.00**	**0.00**	**0.00**
EldA	−0.001	0.022	^*^	0.90	0.66	0.99	0.99	0.32	0.70	0.69	**0.00**	**0.00**
EldB	−0.018	−0.010	−0.021	^*^	0.12	0.70	0.46	0.37	0.13	**0.00**	**0.00**	**0.00**
Iv Coast	0.025^*^	0.029^*^	−0.012	0.014	^*^	**0.02**	0.75	0.33	0.37	0.71	**0.00**	**0.00**
Crete Is	0.001	0.020	−0.023	−0.010	0.031^*^	^*^	0.13	**0.00**	**0.02**	**0.00**	**0.00**	**0.00**
Basq C	0.009	0.023^*^	−0.040	−0.001	−0.004	0.011	^*^	**0.04**	0.14	**0.04**	**0.00**	**0.00**
Siwa O	0.010	0.001	0.011	0.004	0.003	0.031^*^	0.013^*^	^*^	**0.00**	**0.00**	**0.00**	**0.00**
M H Atlas	0.034^*^	0.043^*^	−0.009	0.021	0.002	0.023^*^	0.008	0.021^*^	^*^	0.79	**0.00**	**0.00**
Tun	0.038^*^	0.043^*^	−0.011	0.025^*^	−0.005	0.034^*^	0.008^*^	0.017^*^	−0.005	^*^	**0.00**	**0.00**
Ay sp	0.115^*^	0.114^*^	0.350^*^	0.156^*^	0.264^*^	0.141^*^	0.185^*^	0.163^*^	0.229^*^	0.225^*^	^*^	0.34
Q sp	0.138^*^	0.135^*^	0.428^*^	0.207^*^	0.293^*^	0.174^*^	0.207^*^	0.179^*^	**0.255**	0.245^*^	0.001	^*^

Values marked with ^*^ (highlighted in bold) correspond to p-values < 0.05. Pos, Posadas; Cor, Corrientes; EldA, EldoradoA; EldB, EldoradoB; Iv Coast, Ivory Coast; Crete Is, Crete Island; Basq C, Basque Country; Siwa O, Siwa Oasis; M H Atlas, Moroccan High Atlas; Tun, Tunisia; Ay sp, Aymará speakers; Q sp, Quechua speakers.

**Figure 2:**
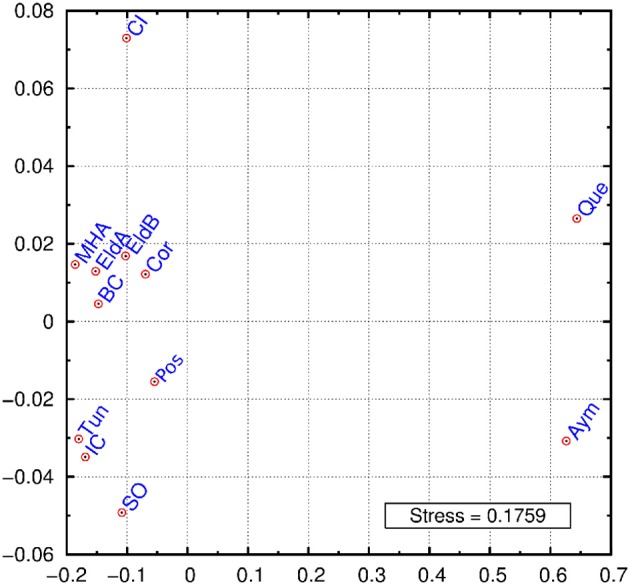
MDS graphic using *Reynolds* index. Eldorado A (EldA), Eldorado B (EldB), Corrientes (Cor), Posadas (Pos), Basque Country (BC), Tunisia (Tun), Ivory Coast (IC), Siwa Oasis (SO), Crete Island (CI), Moroccan High Atlas (MHA), Quechua speakers (Que), Aymará speakers (Aym).

A multidimensional scaling plot (MDS) was graphed employing *Reynolds* index (Figure 2). The first dimension of the graph clearly separates Bolivians from the rest of populations, while the second dimension stresses the differentiation of two samples: Crete Island and Siwa Oasis. The rest of the samples cluster in an intermediate position, our Argentinian samples except Posadas are grouped together with an European and a North African sample. When these populations were grouped in Europeans, Africans, Bolivians and Argentinians the exact test gave signicant differentiation between them (*p* < 0.05) (data not shown) probably as a result of the Bolivian differentiation from one side, and the presence of Siwa Oasis in the African group, and Crete Island in the European one. The outlier position of these two samples may be an artifact due to the relatively low number of loci analyzed. The proximity of Basque data to our populations can be explained given that Argentina is the country with highest rate of Basque immigration in the world (Eusko Jaurlaritza - Gobierno Vasco, 2000; Ezkerro, 2002), while the Moroccan sample similarity might respond to an ancient relationship between northern Africa and southern Spain.

**Figure 3:**
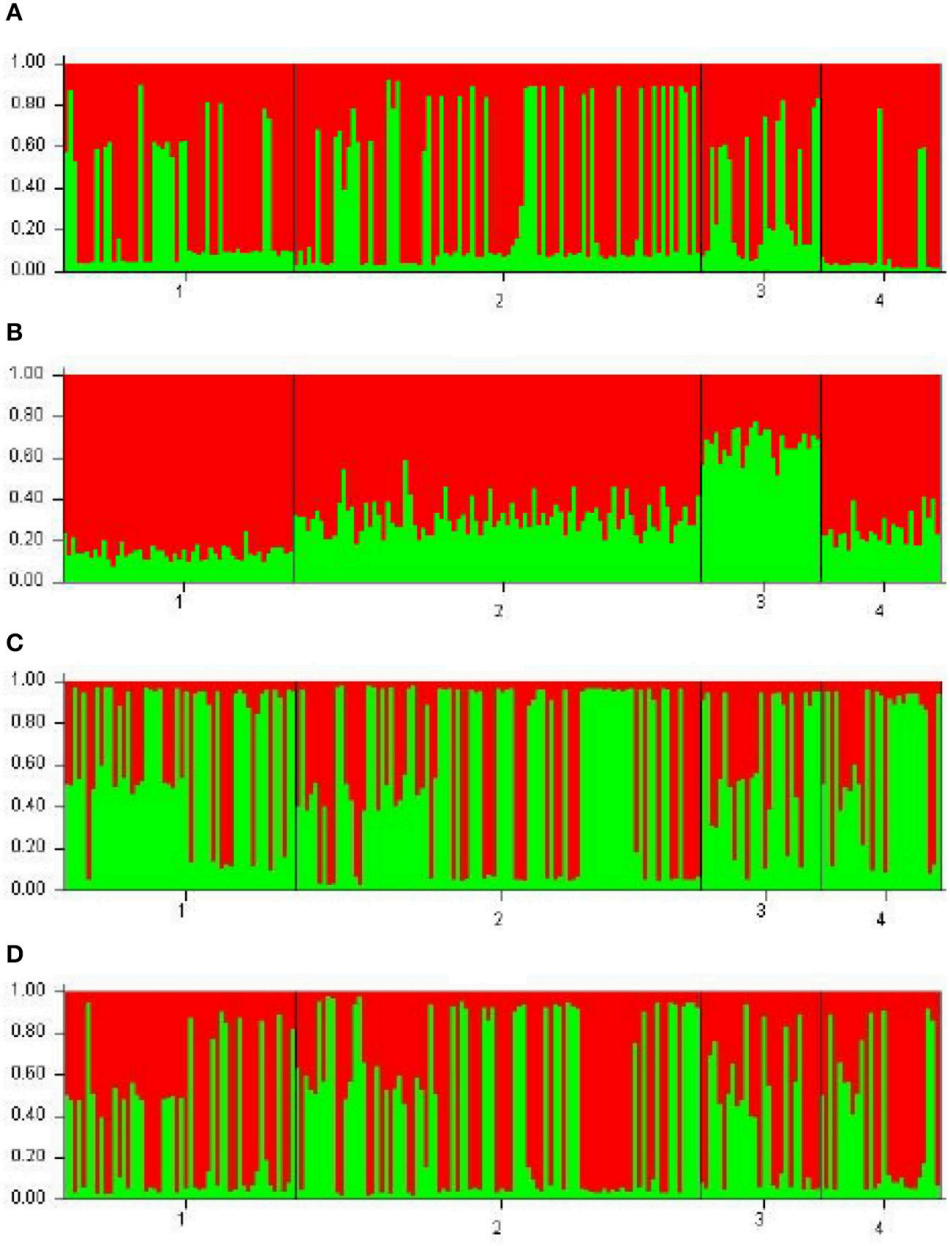
Bar plots using *k* = 2 for the populations of Posadas *n* = 52 (28 females 24 males) (1), Corrientes *n* = 92 (32 females 60 males) (2), Eldorado A *n* = 27 (13 females 14 males) (3), and Eldorado B *n* = 27 (11 females 16 males) (4). **(A)** is for 10 Alu insertions, **(B)** is for the 5 INDELs, **(C)** is for the 15 SNPs, and **(D)** is for the 10 Alu, 5 INDELs, and 15 SNPs.

**Figure 4:**
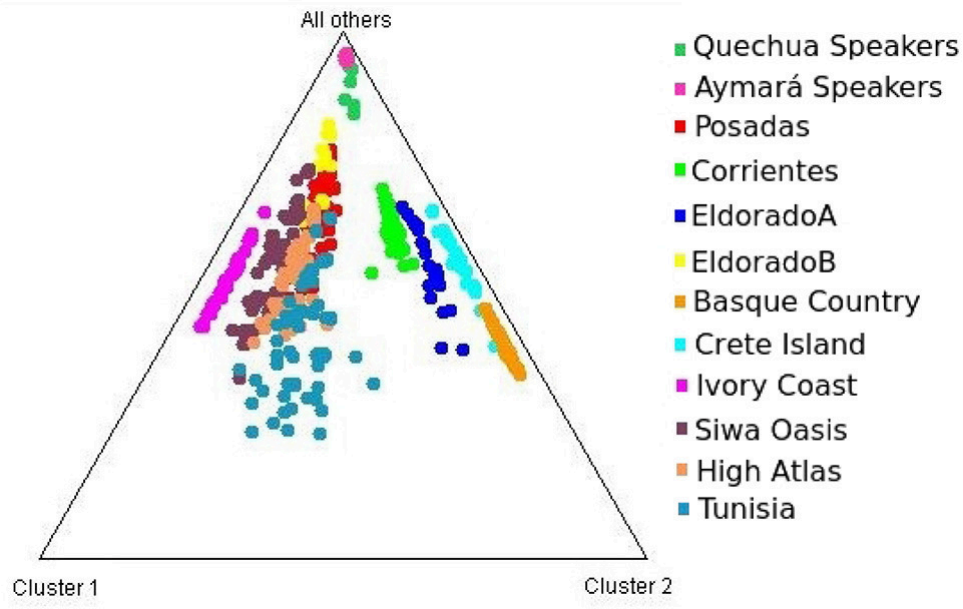
Triangle plot for 8 Alu insertions using *k* = 4 for the populations of Posadas, Corrientes, Eldorado A, Eldorado B, sub-Saharan Africa (Ivory Coast), Moroccan (High Atlas), Siwa Oasis (Egypt), Tunisia, Greece (Crete Island), Spain (Basque Country) and Aymará and Quechua speakers (Bolivia) top of the graphic.

**Figure 5:**
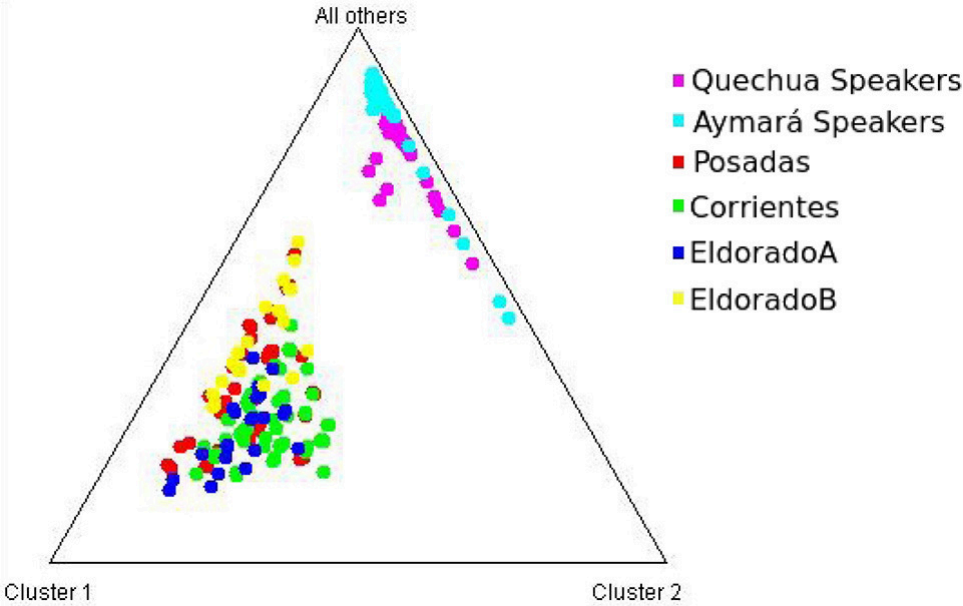
Triangle plot for the 9 Alu insertions using *k* = 3 for the populations of Posadas, Corrientes, Eldorado A, Eldorado B, Quechua speakers, and Aymará speakers.

Each of the analyzed populations presented a different LD pattern. Posadas presented the major LD followed by Corrientes. See Tables S12–S15 for more information. A possible internal structure was analyzed in each of the populations using the Structure software. The results are shown in Figure 3, where similiarity can be observed between Corrientes and Eldorado B populations, probably due to their common origin. In the case of INDELs, they showed a marked interpopulation differentiation of Eldorado A compared to the other populations. *F*_st_ values obtained (Table 3) are in agreement with the differentiation shown with Structure graphics. A comparison against the 8 previously analyzed world populations, assuming the same parameters, is shown in Figures 4, 5. In both graphics a clear differentiation of Bolivian Native populations to any other can be observed. In Figure 4, a close relationship among Eldorado A and the populations of European origin is shown, while Eldorado B seems to share much less European genetic background. Finally, the forensic parameters *PIC, PE, PDf*, and *PDm* were calculated using the 30 markers for the analyzed populations. See Tables S16–S18. The highest values for the SNP markers for Corrientes were rs1299087, rs933315 and rs1781104, for Posadas were rs5986751, rs2209420, and rs933315, for Eldorado A were rs5986751, rs1991961, and rs1781104 and for Eldorado B were rs5964206, rs933315, rs1991961, rs1781104, and rs149910. In the case of INDELs markers the highest values for Corrientes were MID 3756 and MID193, for Posadas were MID193 and MID1540, for Eldorado A were MID3756 and MID1540, and for Eldorado B were MID3754 and MID1705. Finally for Alu markers the highest values for Corrientes and Posadas were Yb8NBC102 and Ya5DP62, for Eldorado A were Yb8NBC102, Ya5DP62, and Ya5NBC37, and for Eldorado B were Ya5NBC37 and Ya5DP77.

## 4. Discussion

In this work we analyzed the differentiation among the populations from three different north-east localities from Argentina: the two capital cities of Posadas and Corrientes, and the city of Eldorado. Previous studies include autosomal SNPs within coding regions for Corrientes city (Lopez-Soto and Catanesi, 2015) and STRs of X chromosome for Corrientes and Posadas (Glesmann et al., 2011). The report on autosomal SNPs showed clear differences in the comparison of Corrientes to other populations of the world, while the report on X-chromosome STRs presented certain differentiation between Corrientes and Posadas (Di Santo et al., 2015). Information on uniparental variability is scarce, although some preliminary data on Corrientes city showed a high component of Amerindian mitochondrial DNA and a very low proportion of native Y chromosome. Given the pattern of inheritance of X-chromosome variation, females can influence the X variation of current populations double than males, thus we can interpret that an important native component is present in X-chromosome variation at this location (Golpe et al., 2017a,b).

The genetic diversity found in this work between the four populations (Misiones, Corrientes, and the two populations of El Dorado) was similar to those referenced in the bibliography (Callinan et al., 2003; Athanasiadis et al., 2007; Gayà-vidal et al., 2009; Rocañín-Arjó et al., 2013), except for two Alu insertions that were monomorphic in some cases, and for the Alu insertion Ya5DP13 that were invariant, similar to previous data for European populations (Callinan et al., 2003; Athanasiadis et al., 2007). According to INDELs results (Figure 2B), the population from Eldorado was clearly separated into two subpopulations (dubbed A and B), as reflected in the number of ancestral populations obtained (*k* = 2). Figure 2B also marked a separation between Eldorado A, and Posadas and Corrientes; this differentiation was also evident in the rest of the comparisons. Consistently, Eldorado A participants reported a specific European ancestry, while Posadas, Corrientes, and Eldorado B participants reported to come from scattered places of Europe, or to have an unknown origin. Thus, a possible endogamy process within Eldorado A could be suggested. Such a process has been observed in other communities of immigrants in which the individuals tend to marry with partners who share their cultural customs (Junta de Estudios Históricos del Municipio de Eldorado, 2016). However, Eldorado A showed the lowest LD, although this result could be biased by the small size of the sample. It is less clear how to explain the high LD results obtained for the Posadas population, as this capital city was expected to receive a considerable migratory flow, in agreement with the average OH value being slightly (though not significantly) higher than the EH. This feature could be the product of a possible internal structure not detected with the HW analysis, therefore collecting further data from this population might help to clarify this issue. The four populations studied did not show high differentiation between them for the Alu insertions variation, reflecting that they share, at least partially, a common origin in the European immigration. This absence of marked interpopulational differences is in accordance with the lower rate of mutation of Alu insertions compared to other markers useful for analyzing the relationship between populations further in time (see e.g., Callinan et al., 2003; Athanasiadis et al., 2007; Gayà-vidal et al., 2009, for structure analysis of populations from other continents and/or countries).

Concerning intercontinental comparisons, results clearly indicated that the Eldorado A population was closer to Europeans. Such higher European ancestry is consistent with the self-reported origin of the participants, whom in most cases answered that their parents or grandparents came from Switzerland or Germany. Comparisons to data from sub-Saharan Africa (Ivory Coast) resulted in its separation from our four populations. This feature might be indicative of a low rate of slave traffic in the past in this region, unlike the more significant flow of slaves occurred across the central and north-west regions of Argentina (Morales and Alfaro, 2000).

A notable differentiation was found between the geographically closer Native Americans from Bolivia and our four populations (Figures 4, 5). The differences might be partially caused by the force of genetic drift acting on the Native groups all along the South American continent generating an exceptional genetic variation on every Native community (Cavalli-Sforza et al., 1996; Zago et al., 1996). There are several examples of Native communities subjected to a considerable reduction of variability as a result of isolation and small size, as in the case of Gran Chaco (Catanesi et al., 2007; Glesmann et al., 2013) and Amazonia Native people (Zago et al., 1996). But genetic drift was not the only process which increased the separation among different tribes. On the one hand, in the past the Guaraní predominated in North-east Argentina. Between 1609 and 1767 they were introduced to Jesuit evangelization missions, generating an admixed context which integrated them into the European civilization. At the beginning of the evangelization process the Guaraní were separated from the Spanish in full-service towns, and a technical organization was promoted by Jesuits. The rights of the Guaraní people were progressively guaranteed, favoring their population growth. After the priests of the Jesuit community were dismissed from South America, the Guaraní, instead of returning to the forest, started integrating with the European colonies, giving rise to the current admixed population. Nowadays, some Guaraní communities remain in the Misiones province, but those from the Corrientes province gradually lost their seminomadic habits and their identity, and finally integrated with the urban populations (Heguy, 2012). On the other hand, the Bolivian Andes region was included in the Inca empire, which reached its limit in north-eastern Gran Chaco when the incas faced the native warriors living there (Ibanez, 2008). For that reason, it is reasonable to find a differentiation between Aymará-Quechua and communities from North-east Argentina, since the latter were involved in a process called Guaranization, and there currently exists a considerable component of Guaraní of Mbya origin that is neither Aymará nor Quechua (Magrassi, 1989). The X chromosome binary markers used in this work proved to be informative for differentiating the populations from North-east Argentina, where different levels of admixture with Native people could be observed. Future analysis including a wider range of X chromosome markers in new sampled individuals will surely clarify some unresolved aspects. So far, the Alu insertions resulted useful for comparing distantly related populations, as they gave important information for distinguishing a clear European origin in Eldorado A. Concerning INDELs and SNPs variation, they were more informative for revealing the differentiation within North-east Argentina, as expected.

## Author contributions

GD and CC: study design; CA and IA: data collection; GD and SdP: data processing; GD, CC, and ME: data analysis; GD, SdP, and CC: manuscript preparation.

### Conflict of interest statement

The authors declare that the research was conducted in the absence of any commercial or financial relationships that could be construed as a potential conflict of interest.
