# Supplementary material for: Genetic Differentiation of North-East Argentina Populations Based on 30 Binary X Chromosome Markers
Source: Front Genet. 2018 Jun 13;9:208. doi: 10.3389/fgene.2018.00208 (PMC6008373; doi:10.3389/fgene.2018.00208)
Supplement: Supplementary file 1 [file Table_1.PDF]

Supplementary Material:
Genetic differentiation of North-east Argentina populations based on 30 binary X chromosome markers

Di Santo Meztler, G. P. [1,*], **del Palacio, S.** [2], **Esteban, M. E.** [3,4], **Armoa, I.** [5], **Argüelles, C.** [6], **and Catanesi, C. I.** [1,7]
*Correspondence:
Di Santo Meztler, G. P.
unlpbiotec@gmail.com

1 SUPPLEMENTARY DATA

For Alu markers, the PCR reaction was carried out in a final volume of 10 μl where the reaction mixture was as follows:

- 1X buffer (Inbio Highway®)
- 2 mM of $MgCl_2$
- Specific primers, quantities detailed in Table S2
- 0.025 U of Taq ADN polymerase
- 20 ng of DNA
- H_2O needed to complete 10 μl

Tables S1 and S2 show the conditions of the cyclings and the concentrations of the reagents for each Alu polymorphism.

Steps	T [C]	Time
**Initial denaturation**	94	2'30"
**Denaturation**	94	1'
**Annealing**	Variable	1'
**Extension**	72	1'
**Final extension**	72	10'
**Cycles**	32	

Table S1. Base cycling for 10 X-Alu.

For SNP, the PCR reaction was carried out in a final volume of 10 μl where the reaction mixture was as follows:

- 1X buffer (Inbio Highway®.)
- from 1.8 to 2 mM of $MgCl_2$
- Specific primers, quantities specified in Table S3.

**Alu**	Yb8NBC634(1) - Ya5DP77(2)	Ya5DP4(1) - Ya5NBC491(2)	Ya5NBC37	Yb8NBC102
T_a **[C]**	54	56	60	60
**PC1** [pmol]	20	25	25	25
**PC2** [pmol]	25	25	–	–
**TD**	None	1 C - 56 C - 5 cycles	None	None
**Alu**	Yb8DP49	Ya5DP3	Ya5DP62	Ya5DP13
T_a **[C]**	56	54	54	58
**PC1** [pmol]	25	25	25	25
**PC2** [pmol]	–	–	–	–
**TD**	None	None	None	None

Table S2. Concentration of the primers in the mix and details of the cyclings of each marker. TD = *touch down*; T_a = Temperature of annealing; PC1= Primer Concentration 1, PC2= Primer Concentration 2.

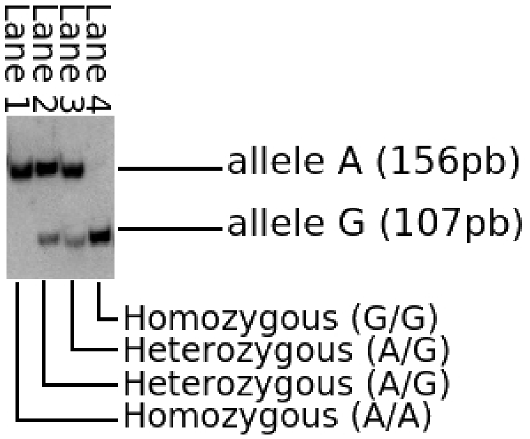

Figure S1. Polyacrylamide gel electrophoresis of SNP rs1299087 fragments cut with restriction enzyme MSPI. Lane 1: homozygote A, lanes 2 and 3: heterozygote, lane 4: homozygote G.

- 0.025 U of Taq ADN polymerase
- from 5 to 30 ng of DNA
- H_2O needed to complete 10 μl

Table S3 shows the conditions of the cyclings and the concentrations of the reagents for each SNP polymorphism.

SNPs										
**Steps**	**rs149910**		**rs1299087**		**rs1781104**		**rs1991961**		**rs652**	
	T [C]	Time	T [C]	Time	T [C]	Time	T [C]	Time	T [C]	Time
**Initial denaturation**	94	2’	94	2’	94	2’	94	2’	94	2’
**Denaturation**	94	45”	94	45”	93	40”	93	45”	93	45”
**Annealing**	54	1’	54	1’	59	1’	64	1’	66	1’
**Extension**	72	1’	72	1’	72	1’	72	1’	72	1’
**Final extension**	72	5’	72	5’	72	10’	72	5’	72	5’
**Cycles**	34		34		34		34		34	
**TD**	None		None		1C/56C/05 cycles		1C/60C/02 cycles		1C/62C/02 cycles	
**PC1**	25 pmol		30 pmol		25 pmol		35 pmol		14 pmol	
$MgCl_2$	2 mM		2 mM		2 mM		2 mM		2 mM	
**Quantity of DNA**	20 ng		25 ng		25 ng		30 ng		20 ng	
**CRE**	HinfI		MSPI		None		None		None	
**Steps**	**rs5964206(1) rs318173(2)**		**rs2209420(1) rs6639398(2)**		**rs9781645(1) rs933315(2)**		**rs4825889(1) rs1937193(2) / rs1781116(3)**		**rs5986751**	
	T [C]	Time	T [C]	Time	T [C]	Time	T [C]	Time	T [C]	Time
**Initial denaturation**	94	2’	94	2’	94	2’	94	2’	94	2’
**Denaturation**	93	40”	93	40”	93	40”	93	40”	93	40”
**Annealing**	60	55”	60	55”	61	55”	62	55”	59	55”
**Extension**	72	1’	72	1’	72	1’	72	1’	72	1’
**Final extension**	72	10’	72	10’	72	10’	72	10’	72	10’
**Cycles**	34		34		34		34		34	
**TD(touch down)**	1C/58C/05 cycles		1C/58C/05 cycles		1C/61C/05 cycles		1C/60C/05 cycles		1C/56C/05 cycles	
**PC1**	28 pmol		35 pmol		30 pmol		25 pmol		12 pmol	
**PC2**	28 pmol		25 pmol		30 pmol		25 pmol		1.8 mM	
**PC3**	–		–		–		25pmol		–	
$MgCl_2$	2 mM		2 mM		2 mM		2 mM		–	
**Quantity of DNA**	20 ng		20 ng		25 ng		25 ng		5-7 ng	
**CRE**	None		None		None		None		None	

Table S3. Concentration of the primers in the mix and details of the cyclings of each marker. PC1= Primer Concentration 1, PC2= Primer Concentration 2, PC3= Primer Concentration 3. **CRE=** cutting restriction enzyme.

Marker	Primers
**rs1781104**	FwA: TGTCTTGTCCAATGCCGAGA<br>FwC: TGTCTTGTCCAATGCCTAGC<br>Rv: GGCAAATTGACTGGAAGCCA
**rs1781116**	FwT: GCAAATATGTTGGCAAGACGTAT<br>FwC: GCAAATATGTTGGCAAGACATAC<br>Rv: CCCTTCCCCACCTTGGATAG
**rs318173**	Fw: CATCGCCCAAGGAGTGATATA<br>RvA: TCTGGGTATATTCAGAAGCA<br>RvC: CTCTGGGTATATTCAGAAGCC
**rs4825889**	Fw: TCTTTGGCTCCCCTAAATTGCT<br>RvA: ATGAACCGAGTTTTGATTTGGAAT<br>RvC: ATGAACCGAGTTTTGATTTGAAAC
**rs933315**	FwA: TGGGAAGACCAAAAACAAGCAA<br>FwG: TGGGAAGACCAAAAACAATCAG<br>Rv: TGCCCCACAGAGATTTTAGTG
**rs1991961**	Fw: GTACGAGATGACTGACCACCA<br>RvG: CTGACTAAGTGGAGACAACCG<br>RvT: CTGACTAAGTGGAGACACCCT
**rs1937193**	Fw: GTCTCTCATATCCAGTGTAGGT<br>RvT: TGCCTCAAACTCTTTCTTCTCAT<br>RvA: TGCCTCAAACTCTTTCTTCACAA
**rs652**	FwG: TGTCCCACACCTACAGTGCG<br>FwT: TGTCCCACACCTACATTGCT<br>Rv: ACCTGTGCATGCATTTGTGTAC
**rs6639398**	FwA: TCAGGACAGAGAGACATGTGTA<br>FwG: TCAGGACAGAGAGACATTTGTG<br>Rv: CTCATGCTCAGTACCATCTTGT
**rs5986751**	FwC: TTTTGCTTCCCTCACTGTGC<br>FwT: TTTTGCTTCCCTCACGGTGT<br>Rv: TTATGTTCCCTCAGCCCAGA
**rs5964206**	FwC: TGTCCTTCATCAGAAAAAATTCTC<br>FwT: TGTCCTTCATCAGAAAAAAGTCTT<br>Rv: CAACACGGCTCTATATCACTGA
**rs2209420**	FwA: CTCTTGTAACCACTATTTGATTCA<br>FwC: CTCTTGTAACCACTATTTAATTCC<br>Rv: AGTGAACTCAGTGGATAAATGGA
**rs9781645**	FwC: TGTCCTTCATCAGAAAAAATTCTC<br>FwT: TGTCCTTCATCAGAAAAAAGTCTT<br>Rv: CAACACGGCTCTATATCACTGA
**rs149910**	Fw:GAAGATTTACTCAAATAGAGTG<br>Rv: TCCATTGTTCATTTCAAGGAAT<br>**CRE Hinf I**
**rs1299087**	Fw: TTTTAGAGGGATTCAGACACAAA<br>Rv: CGCTTTGCAATTGGTTGTAT<br>**CRE MSP I**

Table S4. Primer sequences for the 15 SNPs. **CRE=** cutting restriction enzyme.

The supplementary information for genotypic frequencies, observed heterozygosity (OH), expected heterozygosity (EH), Hardy-Weinberg equilibrium (HW) for females, linkage disequilibrium (LD) for males and forensic parameters are presented in Tables S5 to S18.

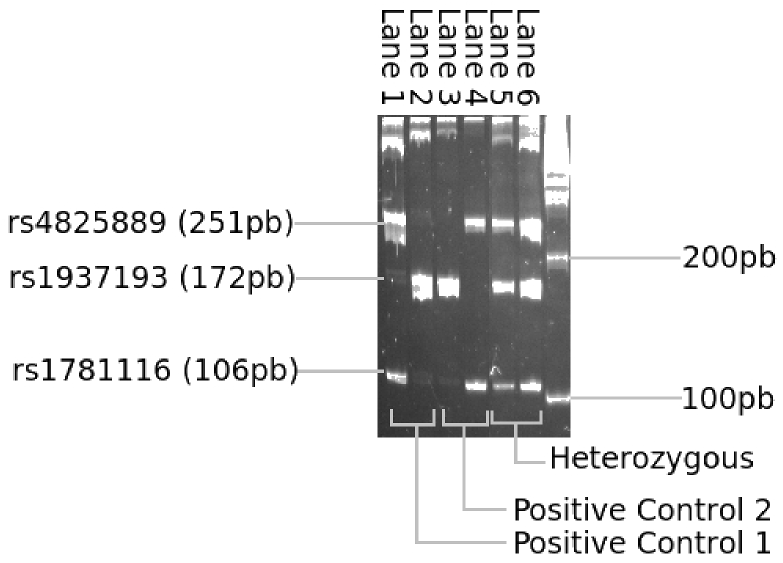

	Positive 1		Positive 2		Heterozygous S.	
Marker	Lane 1	Lane 2	Lane 3	Lane 4	Lane 5	Lane 6
rs4825889	C	-	-	T	C	T
rs1937193	-	T	A	-	A	T
rs1781116	T	-	-	C	T	C

Figure S2. Polyacrylamide gel electrophoresis of SNPs rs4825889, rs1937193 and rs1781116 amplified in a multiplex reaction using allele specific primers. For each sample, two different contiguous lanes show the presence or absence of the alleles corresponding to the 3 SNPs.

Marker	Genotype	Corrientes	Posadas	EldoradoA	EldoradoB
**Ya5DP3**	0-0	0.83	0.68	0.77	1
	0-1	0.17	0.29	0.23	0
	1-1	0	0.04	0	0
**Ya5DP4**	0-0	0.97	1	1	1
	0-1	0.03	0	0	0
	1-1	0	0	0	0
**Ya5 491**	0-0	0	0	0	0
	0-1	0.03	0.04	0	0
	1-1	0.97	0.96	1	1
**Ya5DP13**	0-0	0	0	0	0
	0-1	0	0	0	0
	1-1	1	1	1	1
**Yb8NBC634**	0-0	0	0	0.08	0
	0-1	0.03	0.07	0	0
	1-1	0.97	0.93	0.92	1
**Yb8NBC102**	0-0	0.16	0.07	0	0
	0-1	0.19	0.43	0.42	0.18
	1-1	0.66	0.50	0.58	0.82
**Ya5DP62**	0-0	0.13	0.07	0	0
	0-1	0.26	0.43	0.25	0.18
	1-1	0.61	0.50	0.75	0.82
**Ya5NBC37**	0-0	0.62	0.64	0.45	0.55
	0-1	0.31	0.36	0.45	0.27
	1-1	0.06	0	0.09	0.18
**Yb8DP49**	0-0	0.03	0	0.11	0
	0-1	0.13	0.14	0.33	0.18
	1-1	0.83	0.86	0.56	0.82
**Ya5DP77**	0-0	0.10	0.11	0.10	0.09
	0-1	0.52	0.43	0.10	0.45
	1-1	0.39	0.46	0.80	0.45

Table S5. Genotypic frequencies of the populations of Corrientes (n=32 females), Posadas (n=28 females), Eldorado A (n=13 females) and Eldorado B (n=11 females). Allele 0 corresponds to the deletion while allele 1 corresponds to the insertion.

Marker	Genotype	Corrientes	Posadas	Eldorado A	Eldorado B
**rs6639398**	GG	0.68	0.61	0.46	0.55
	AG	0.29	0.36	0.46	0.45
	AA	0.03	0.04	0.08	0
**rs5986751**	TT	0.39	0.22	0.36	0.27
	TC	0.52	0.52	0.27	0.27
	CC	0.10	0.26	0.36	0.45
**rs5964206**	GG	0.19	0.07	0	0.36
	GC	0.34	0.54	0.08	0.27
	CC	0.47	0.39	0.92	0.36
**rs9781645**	TT	0.53	0.14	0.12	0.60
	TC	0.27	0.43	0.50	0.40
	CC	0.20	0.43	0.38	0
**rs2209420**	CC	0.10	0.25	0.11	0
	CA	0.42	0.32	0.33	0.36
	AA	0.48	0.43	0.56	0.64
**rs1299087**	GG	0.33	0.50	0.20	0.18
	GA	0.43	0.32	0.60	0.55
	AA	0.23	0.18	0.20	0.27
**rs318173**	CC	0	0.07	0.17	0.18
	CA	0.28	0.43	0.50	0.18
	AA	0.72	0.50	0.33	0.64
**rs933315**	GG	0.20	0.26	0	0.27
	GA	0.40	0.56	0.25	0.36
	AA	0.40	0.19	0.75	0.36
**rs1991961**	TT	0.40	0.32	0.14	0.36
	GT	0.47	0.36	0.57	0.36
	GG	0.13	0.32	0.29	0.27
**rs4825889**	TT	0.31	0.46	0.30	0.30
	TC	0.56	0.50	0.60	0.70
	CC	0.12	0.04	0.10	0
**rs1781116**	TT	0.19	0.04	0.10	0.10
	TC	0.50	0.50	0.60	0.60
	CC	0.31	0.46	0.30	0.30
**rs1937193**	TT	0.19	0.04	0.10	0.10
	TA	0.50	0.54	0.60	0.60
	AA	0.31	0.43	0.30	0.30
**rs1781104**	CC	0.47	0.04	0.38	0.27
	CA	0.28	0.68	0.62	0.55
	AA	0.25	0.29	0	0.18
**rs149910**	TT	0.16	0.27	0.09	0.09
	TG	0.52	0.42	0.73	0.64
	GG	0.32	0.31	0.18	0.27
**rs652**	TT	0.12	0.18	0.10	0.36
	TG	0.44	0.50	0.30	0.45
	GG	0.44	0.32	0.60	0.18

Table S6. Genotypic frequencies of the populations of Corrientes (n=32 females), Posadas (n=28 females), Eldorado A (n=13 females) and Eldorado B (n=11 females).

Marker	Genotype	Corrientes	Posadas	EldoradoA	EldoradoB
**MID3754**	0-0	0.23	0.04	0.08	0.45
	0-1	0.52	0.74	0.23	0.09
	1-1	0.26	0.22	0.69	0.45
**MID3756**	0-0	0.25	0.11	0.38	0.55
	0-1	0.53	0.50	0.46	0.36
	1-1	0.22	0.39	0.15	0.09
**MID1705**	0-0	0	0.18	0	0.20
	0-1	0.40	0.46	0.31	0.60
	1-1	0.60	0.36	0.69	0.20
**MID193**	0-0	0.32	0.29	0	0.10
	0-1	0.35	0.54	0.50	0.60
	1-1	0.32	0.18	0.50	0.30
**MID1540**	0-0	0.13	0.24	0.38	0.30
	0-1	0.40	0.52	0.38	0.50
	1-1	0.47	0.24	0.23	0.20

Table S7. Genotypic frequencies of the populations of Corrientes (n=32 females), Posadas (n=28 females), Eldorado A (n=13 females) and Eldorado B (n=11 females). Allele 0 corresponds to the deletion while allele 1 corresponds to the insertion.

Marker	Obs Het	Exp Het	p-value*
**Ya5DP3**	0.29	0.30	1.00
**rs6639398**	0.36	0.34	1.00
**Ya5491**	0.04	0.04	1.00
**rs5986751**	0.52	0.51	1.00
**rs5964206**	0.54	0.46	0.42
**rs9781645**	0.43	0.47	0.70
**rs2209420**	0.32	0.49	0.11
**rs1299087**	0.32	0.46	0.20
**Yb8NBC634**	0.07	0.07	1.00
**rs318173**	0.43	0.42	1.00
**rs933315**	0.56	0.51	0.71
**Yb8NBC102**	0.43	0.42	1.00
**Ya5DP62**	0.43	0.42	1.00
**MID3754**	0.74	0.49	0.01
**MID3756**	0.50	0.47	1.00
**Ya5NBC37**	0.36	0.30	0.55
**rs1991961**	0.36	0.51	0.14
**rs4825889**	0.50	0.42	0.38
**rs1781116**	0.50	0.42	0.38
**rs1937193**	0.54	0.43	0.37
**rs1781104**	0.68	0.48	0.04
**rs149910**	0.42	0.51	0.45
**rs652**	0.50	0.50	1.00
**MID1705**	0.46	0.49	1.00
**Yb8DP49**	0.14	0.14	1.00
**Ya5DP77**	0.43	0.44	1.00
**MID193**	0.54	0.50	1.00
**MID1540**	0.52	0.51	1.00

Table S8. OH, EH, and HW for females from Posadas (n=28). Monomorphic markers were not included in the analysis. $^*sd < 0.001$.

Marker	Obs Het	Exp Het	p-value*
**Ya5DP3**	0.17	0.16	1.00
**Ya5DP4**	0.03	0.03	1.00
**rs6639398**	0.29	0.30	1.00
**Ya5491**	0.03	0.03	1.00
**rs5986751**	0.52	0.47	0.70
**rs5964206**	0.34	0.47	0.24
**rs9781645**	0.27	0.45	0.04
**rs2209420**	0.42	0.43	1.00
**rs1299087**	0.43	0.50	0.48
**Yb8NBC634**	0.03	0.03	1.00
**rs318173**	0.28	0.25	1.00
**rs933315**	0.40	0.49	0.45
**Yb8NBC102**	0.19	0.38	0.01
**Ya5DP62**	0.26	0.39	0.08
**MID3754**	0.52	0.51	1.00
**MID3756**	0.53	0.51	1.00
**Ya5NBC37**	0.31	0.35	0.61
**rs1991961**	0.47	0.47	1.00
**rs4825889**	0.56	0.49	0.47
**rs1781116**	0.50	0.50	1.00
**rs1937193**	0.50	0.50	1.00
**rs1781104**	0.28	0.48	0.03
**rs149910**	0.52	0.49	1.00
**rs652**	0.44	0.46	1.00
**MID1705**	0.40	0.33	0.56
**Yb8DP49**	0.13	0.18	0.24
**Ya5DP77**	0.52	0.47	0.70
**MID193**	0.35	0.51	0.15
**MID1540**	0.40	0.45	0.68

Table S9. OH, EH, and HW for females of Corrientes (n=32). Monomorphic markers were not included in the analysis.$^*sd < 0.001$.

Marker	Obs Het	Exp Het	p-value*
**Ya5DP3**	0.23	0.21	1.00
**rs6639398**	0.46	0.44	1.00
**rs5986751**	0.27	0.52	0.22
**rs5964206**	0.08	0.08	1.00
**rs9781645**	0.50	0.50	1.00
**rs2209420**	0.33	0.42	1.00
**rs1299087**	0.60	0.53	1.00
**Yb8NBC634**	0.00	0.15	0.04
**rs318173**	0.50	0.51	1.00
**rs933315**	0.25	0.23	1.00
**Yb8NBC102**	0.42	0.34	1.00
**Ya5DP62**	0.25	0.23	1.00
**MID3754**	0.23	0.32	0.37
**MID3756**	0.46	0.49	1.00
**Ya5NBC37**	0.45	0.45	1.00
**rs1991961**	0.57	0.53	1.00
**rs4825889**	0.60	0.51	1.00
**rs1781116**	0.60	0.51	1.00
**rs1937193**	0.60	0.51	1.00
**rs1781104**	0.62	0.44	0.24
**rs149910**	0.73	0.52	0.26
**rs652**	0.30	0.39	0.48
**MID1705**	0.31	0.27	1.00
**Yb8DP49**	0.33	0.42	1.00
**Ya5DP77**	0.10	0.27	0.16
**MID193**	0.50	0.39	0.53
**MID1540**	0.38	0.51	0.57

Table S10. OH, EH, and HW for females of Eldorado A ($n = 13$). Monomorphic markers were not included in the analysis.$*sd < 0.001$.

Marker	Obs Het	Exp Het	p-value*
**rs6639398**	0.45	0.37	1.00
**Ya5491**	0.27	0.51	0.21
**rs5986751**	0.27	0.52	0.22
**rs9781645**	0.40	0.34	1.00
**rs2209420**	0.36	0.31	1.00
**rs1299087**	0.55	0.52	1.00
**rs318173**	0.18	0.42	0.11
**rs933315**	0.36	0.52	0.54
**Yb8NBC102**	0.18	0.17	1.00
**Ya5DP62**	0.18	0.17	1.00
**MID3754**	0.09	0.52	0.01
**MID3756**	0.36	0.42	1.00
**Ya5NBC37**	0.27	0.45	0.23
**rs1991961**	0.36	0.52	0.54
**rs4825889**	0.70	0.48	0.22
**rs1781116**	0.60	0.51	1.00
**rs1937193**	0.60	0.51	1.00
**rs1781104**	0.55	0.52	1.00
**rs149910**	0.64	0.51	0.55
**rs652**	0.45	0.51	1.00
**MID1705**	0.60	0.53	1.00
**Yb8DP49**	0.18	0.17	1.00
**Ya5DP77**	0.45	0.45	1.00
**MID193**	0.60	0.51	1.00
**MID1540**	0.50	0.52	1.00

Table S11. OH, EH, and HW for females of Eldorado B ($n = 11$). Monomorphic markers were not included in the analysis.$*sd < 0.001$.

	0	1	2	5	6	7	8	9	10	11	12	13	14	15	16	17	18	19	20	21	22	23	24	25	26	27	28	29
**0**	*	1.000	1.000	0.136	1.000	0.100	0.019	0.406	1.000	1.000	1.000	0.564	0.509	0.067	0.292	0.513	0.558	0.079	0.053	0.053	0.198	0.104	0.321	1.000	1.000	1.000	0.282	0.035
**1**	0.002	*	1.000	1.000	1.000	1.000	1.000	1.000	1.000	1.000	1.000	1.000	1.000	1.000	1.000	1.000	1.000	1.000	1.000	1.000	1.000	1.000	1.000	1.000	1.000	1.000	1.000	1.000
**2**	0.017	0.002	*	0.521	0.128	0.069	0.118	0.737	1.000	1.000	0.476	0.363	0.351	0.086	0.174	0.482	0.312	0.247	0.280	0.280	0.079	0.079	0.260	0.152	1.000	1.000	0.172	0.077
**5**	0.003	0.012	0.056	*	0.237	0.022	0.469	0.108	0.162	0.725	0.044	0.012	0.018	0.533	0.099	0.147	0.075	0.011	0.054	0.054	0.105	0.106	0.424	0.229	0.554	0.282	0.064	0.107
**6**	0.067	0.036	0.001	0.019	*	0.287	0.252	0.061	0.256	0.174	0.097	0.183	0.211	0.333	0.012	0.219	0.000	0.051	0.113	0.113	0.002	0.435	0.154	0.469	0.189	0.150	0.200	0.050
**7**	0.002	0.022	0.001	0.000	0.048	*	0.023	0.120	1.000	0.022	0.280	0.144	0.144	0.205	0.025	0.038	0.056	0.127	0.083	0.083	0.090	0.010	0.100	0.036	0.154	0.057	0.090	0.147
**8**	0.000	0.035	0.004	0.081	0.019	0.000	*	0.118	0.229	0.314	0.398	0.053	0.070	0.024	0.041	0.242	0.491	0.432	0.459	0.459	0.545	0.103	0.442	0.388	0.157	1.000	0.249	0.053
**9**	0.025	0.022	0.091	0.006	0.002	0.014	0.009	*	0.094	0.009	0.104	0.141	0.129	0.040	0.142	0.040	0.054	0.176	0.212	0.212	0.280	0.044	0.620	0.186	0.138	0.681	0.188	0.087
**10**	0.004	0.001	0.005	0.001	0.005	0.031	0.004	0.000	*	0.408	1.000	1.000	1.000	1.000	1.000	0.293	0.197	1.000	0.171	0.171	0.065	1.000	1.000	0.378	1.000	1.000	0.050	0.035
**11**	0.022	0.003	0.026	0.067	0.011	0.000	0.036	0.000	0.032	*	0.720	0.322	0.333	0.704	0.069	0.242	0.108	0.121	0.079	0.079	0.045	0.129	0.367	0.309	0.028	0.079	0.156	0.276
**12**	0.095	0.015	0.040	0.002	0.006	0.054	0.072	0.007	0.030	0.070	*	0.097	0.083	0.348	0.071	0.078	0.164	0.054	0.015	0.015	0.052	0.097	0.400	0.136	0.018	0.398	0.032	0.074
**13**	0.021	0.036	0.037	0.000	0.008	0.009	0.001	0.012	0.017	0.009	0.006	*	1.000	0.246	0.060	0.087	0.188	0.055	0.094	0.094	0.099	0.012	0.009	0.070	0.737	0.150	0.100	0.111
**14**	0.016	0.036	0.034	0.000	0.011	0.009	0.001	0.010	0.018	0.010	0.010	1.000	*	0.273	0.053	0.118	0.136	0.000	0.046	0.046	0.129	0.027	0.018	0.054	0.737	0.143	0.100	0.142
**15**	0.000	0.031	0.002	0.119	0.095	0.033	0.000	0.001	0.022	0.057	0.083	0.016	0.022	*	0.222	0.152	0.097	0.202	0.178	0.178	0.095	0.225	0.556	0.250	0.124	0.200	0.252	0.081
**16**	0.008	0.012	0.002	0.009	0.000	0.000	0.001	0.012	0.025	0.001	0.004	0.001	0.001	0.022	*	0.298	0.105	0.184	0.147	0.147	0.143	0.188	0.136	0.270	0.065	0.042	0.064	0.067
**17**	0.015	0.043	0.012	0.006	0.041	0.001	0.012	0.001	0.007	0.005	0.002	0.001	0.003	0.005	0.052	*	0.036	0.095	0.008	0.008	0.104	0.211	0.079	0.242	0.063	0.192	0.007	0.050
**18**	0.024	0.012	0.024	0.005	0.000	0.002	0.002	0.002	0.003	0.004	0.020	0.012	0.006	0.008	0.010	0.000	*	0.277	0.336	0.336	0.238	0.221	0.124	0.097	0.665	0.082	0.053	0.300
**19**	0.001	0.011	0.005	0.000	0.002	0.012	0.060	0.023	0.022	0.002	0.002	0.001	0.000	0.035	0.032	0.002	0.071	*	1.000	1.000	1.000	0.255	0.033	0.099	0.143	0.623	0.095	0.082
**20**	0.001	0.012	0.007	0.001	0.009	0.006	0.073	0.036	0.002	0.002	0.000	0.003	0.001	0.025	0.022	0.000	0.097	0.932	*	1.000	1.000	0.234	0.033	0.058	0.118	0.640	0.136	0.120
**21**	0.001	0.012	0.007	0.001	0.009	0.006	0.073	0.036	0.002	0.002	0.000	0.003	0.001	0.025	0.022	0.000	0.097	0.932	1.000	*	1.000	0.234	0.033	0.058	0.118	0.640	0.136	0.120
**22**	0.006	0.016	0.001	0.007	0.000	0.008	0.139	0.073	0.000	0.000	0.002	0.005	0.010	0.006	0.017	0.005	0.036	0.707	0.759	0.759	*	0.221	0.065	0.107	0.000	0.693	0.123	0.062
**23**	0.003	0.010	0.001	0.009	0.054	0.000	0.003	0.001	0.020	0.005	0.007	0.000	0.001	0.017	0.028	0.011	0.049	0.061	0.047	0.047	0.032	*	0.194	0.260	0.028	0.104	0.050	0.223
**24**	0.004	0.005	0.002	0.066	0.014	0.003	0.003	0.080	0.005	0.082	0.033	0.000	0.000	0.051	0.003	0.004	0.007	0.000	0.000	0.000	0.001	0.016	*	0.746	0.107	0.095	0.222	0.298
**25**	0.035	0.075	0.001	0.018	0.114	0.001	0.017	0.007	0.022	0.075	0.004	0.003	0.002	0.009	0.011	0.036	0.004	0.002	0.001	0.001	0.003	0.026	0.443	*	1.000	0.111	0.483	0.018
**26**	0.032	0.005	0.038	0.054	0.018	0.007	0.012	0.005	0.009	0.001	0.000	0.072	0.072	0.007	0.001	0.000	0.049	0.009	0.006	0.006	0.000	0.000	0.001	0.054	*	1.000	0.423	0.108
**27**	0.017	0.002	0.020	0.016	0.016	0.000	0.068	0.052	0.005	0.000	1.319	0.006	0.006	0.008	0.000	0.012	0.002	0.033	0.037	0.037	0.057	0.003	0.005	0.007	0.037	*	0.457	0.172
**28**	0.008	0.020	0.005	0.002	0.022	0.008	0.033	0.027	0.000	0.005	0.001	0.006	0.006	0.041	0.002	0.000	0.002	0.006	0.013	0.013	0.014	0.002	0.016	0.065	0.040	0.033	*	0.138
**29**	0.000	0.016	0.001	0.009	0.001	0.017	0.002	0.006	0.000	0.014	0.005	0.006	0.010	0.004	0.004	0.001	0.056	0.004	0.010	0.010	0.003	0.035	0.023	0.000	0.003	0.005	0.017	*

Table S12. LD for males of Corrientes ($n = 60$), where **0=** Ya5DP3, **1=** Ya5DP4, **2=** rs6639398, **5=** rs5986751, **6=** rs5964206, **7=** rs9781645,**8=** rs2209420, **9=** rs1299087, **10=** Yb8NBC634, **11**=rs318173, **12=** rs933315, **13=** Yb8NBC102, **14=** Ya5DP62, **15=** MID3754, **16=** MID3756, **17=** Ya5NBC37, **18=** rs1991961, **19=** rs4825889, **20=** rs1781116, **21=** rs1937193, **22=** rs1781104, **23=** rs149910, **24** rs652, **25=** MID1705, **26=** Yb8DP49, **27=** Ya5DP77, **28=** MID193, **29=** MID1540. Above diagonal: D'-values. Below diagonal: r^2-values.

	0	2	5	6	7	8	9	11	12	13	14	15	16	17	18	19	20	21	22	23	24	25	26	27	28	29
**0**	*	1.000	0.333	1.000	0.300	0.333	0.333	0.022	0.022	0.185	0.185	1.000	0.542	0.511	1.000	0.022	0.022	0.067	0.333	0.095	0.022	0.125	0.511	1.000	0.222	1.000
**2**	0.008	*	1.000	1.000	1.000	1.000	1.000	1.000	1.000	1.000	1.000	1.000	1.000	1.000	1.000	1.000	1.000	1.000	1.000	1.000	1.000	1.000	1.000	1.000	1.000	1.000
**5**	0.018	0.044	*	0.429	0.477	0.273	0.000	0.429	0.333	0.500	0.500	0.111	0.000	0.714	0.200	0.250	0.250	0.403	0.167	0.213	0.000	0.071	0.714	0.333	0.164	0.179
**6**	0.074	0.106	0.076	*	0.104	0.065	0.429	0.510	0.619	0.143	0.143	0.238	1.000	0.510	0.314	0.143	0.143	0.179	0.143	0.192	0.357	0.452	0.510	0.059	0.343	0.061
**7**	0.014	0.042	0.228	0.004	*	0.430	0.429	0.403	0.148	1.000	1.000	0.216	0.042	0.042	0.042	0.042	0.042	0.057	0.050	0.500	0.281	0.250	0.452	0.303	0.022	0.057
**8**	0.018	0.051	0.063	0.002	0.155	*	0.129	0.736	0.515	0.455	0.455	0.273	0.692	0.209	1.000	0.077	0.077	0.179	0.091	0.417	0.182	0.303	0.377	0.385	0.164	0.403
**9**	0.018	0.044	0.000	0.076	0.017	0.007	*	0.143	0.111	1.000	1.000	0.111	0.000	0.143	0.600	0.250	0.250	0.403	0.333	0.475	0.000	0.071	0.429	0.333	0.164	0.104
**11**	0.000	0.106	0.076	0.044	0.065	0.264	0.008	*	0.238	1.000	1.000	0.238	0.529	0.020	0.314	0.143	0.143	0.061	0.429	0.250	0.571	0.635	0.020	1.000	0.343	0.179
**12**	0.000	0.026	0.067	0.095	0.015	0.135	0.007	0.014	*	1.000	1.000	0.111	1.000	0.238	0.040	0.400	0.400	0.343	0.111	0.192	0.333	0.432	0.314	0.111	0.115	0.531
**13**	0.024	0.009	0.050	0.002	0.164	0.035	0.200	0.082	0.120	*	1.000	0.200	0.000	0.294	0.368	0.250	0.250	0.179	0.500	0.596	0.625	1.000	0.294	1.000	0.425	1.000
**14**	0.024	0.009	0.050	0.002	0.164	0.035	0.200	0.082	0.120	1.000	*	0.200	0.000	0.294	0.368	0.250	0.250	0.179	0.500	0.596	0.625	1.000	0.294	1.000	0.425	1.000
**15**	0.090	0.073	0.007	0.014	0.023	0.038	0.007	0.014	0.012	0.013	0.013	*	0.556	0.086	0.360	0.333	0.333	0.270	0.111	0.016	0.400	0.452	0.086	0.111	0.017	0.124
**16**	0.124	0.015	0.000	0.137	0.001	0.189	0.000	0.227	0.200	0.000	0.000	0.062	*	0.059	1.000	1.000	1.000	1.000	1.000	0.475	0.000	0.148	0.059	0.111	0.233	0.452
**17**	0.088	0.018	0.210	0.044	0.001	0.021	0.008	0.000	0.014	0.042	0.042	0.005	0.003	*	0.153	0.143	0.143	0.061	0.143	0.563	0.571	0.270	0.020	0.059	0.343	1.000
**18**	0.046	0.011	0.011	0.011	0.000	0.223	0.095	0.011	0.001	0.103	0.103	0.057	0.088	0.015	*	1.000	1.000	1.000	0.200	0.031	0.400	0.014	0.435	1.000	0.292	0.281
**19**	0.000	0.087	0.031	0.017	0.001	0.004	0.031	0.004	0.133	0.006	0.006	0.033	0.167	0.004	0.132	*	1.000	1.000	1.000	0.077	0.250	0.270	0.571	0.000	0.014	0.179
**20**	0.000	0.087	0.031	0.017	0.001	0.004	0.031	0.004	0.133	0.006	0.006	0.033	0.167	0.004	0.132	1.000	*	1.000	1.000	0.077	0.250	0.270	0.571	0.000	0.014	0.179
**21**	0.002	0.104	0.065	0.032	0.001	0.015	0.065	0.001	0.096	0.003	0.003	0.021	0.154	0.001	0.122	1.000	1.000	*	1.000	0.077	0.179	0.185	0.531	0.042	0.014	0.102
**22**	0.018	0.044	0.028	0.008	0.002	0.007	0.111	0.076	0.007	0.050	0.050	0.007	0.333	0.008	0.011	0.500	0.500	0.477	*	0.046	0.250	0.071	0.429	0.333	0.042	0.403
**23**	0.001	0.031	0.025	0.024	0.167	0.080	0.126	0.019	0.037	0.136	0.136	0.000	0.043	0.078	0.001	0.005	0.005	0.005	0.001	*	0.539	0.375	0.308	1.000	0.125	0.121
**24**	0.000	0.087	0.000	0.105	0.046	0.014	0.000	0.067	0.033	0.156	0.156	0.133	0.000	0.067	0.021	0.016	0.016	0.007	0.031	0.236	*	1.000	0.143	0.000	0.425	0.061
**25**	0.002	0.071	0.003	0.112	0.036	0.054	0.003	0.113	0.077	0.328	0.328	0.205	0.005	0.021	0.000	0.021	0.021	0.009	0.003	0.141	0.830	*	0.061	0.148	0.185	0.083
**26**	0.088	0.018	0.210	0.044	0.098	0.049	0.076	0.000	0.068	0.042	0.042	0.005	0.003	0.000	0.121	0.067	0.067	0.054	0.076	0.077	0.017	0.003	*	1.000	0.242	0.521
**27**	0.016	0.006	0.016	0.001	0.013	0.025	0.016	0.059	0.001	0.029	0.029	0.001	0.005	0.001	0.038	0.000	0.000	0.001	0.016	0.081	0.000	0.002	0.059	*	0.410	1.000
**28**	0.006	0.035	0.019	0.040	0.000	0.019	0.019	0.040	0.009	0.029	0.029	0.000	0.015	0.040	0.031	0.000	0.000	0.000	0.002	0.007	0.074	0.016	0.033	0.033	*	0.214
**29**	0.067	0.020	0.015	0.001	0.001	0.065	0.004	0.032	0.192	0.066	0.066	0.013	0.032	0.154	0.038	0.007	0.007	0.002	0.065	0.015	0.0007—	0.002	0.219	0.066	0.026	*

Table S13. LD for males of Posadas ($n = 24$), where **0=** Ya5DP3, **2=** rs6639398, **5=** rs5986751, **6=** rs5964206, **7=** rs9781645,**8=** rs2209420, **9=** rs1299087, **11=**rs318173, **12=** rs933315, **13=** Yb8NBC102, **14=** Ya5DP62, **15=** MID3754, **16=** MID3756, **17=** Ya5NBC37, **18=** rs1991961, **19=** rs4825889, **20=** rs1781116, **21=** rs1937193, **22=** rs1781104, **23=** rs149910, **24** rs652, **25=** MID1705, **26=** Yb8DP49, **27=** Ya5DP77, **28=** MID193, **29=** MID1540. Above diagonal: D'-values. Below diagonal: r^2-values.

	2	5	7	8	9	11	12	13	14	15	16	17	18	19	20	21	22	23	25	26	27	28	29
**2**	*	1.000	1.000	1.000	1.000	1.000	0.083	0.222	0.100	1.000	1.000	0.286	0.083	0.083	0.286	0.111	0.417	1.000	1.000	0.286	1.000	1.000	0.417
**5**	0.400	*	1.000	1.000	1.000	1.000	1.000	0.000	0.250	1.000	1.000	1.000	1.000	0.250	0.250	0.250	0.250	–	–	0.250	1.000	1.000	1.000
**7**	0.375	1.000	*	–	1.000	–	1.000	1.000	1.000	1.000	1.000	–	1.000	1.000	1.000	1.000	1.000	1.000	1.000	1.000	1.000	1.000	1.000
**8**	0.167	1.000	–	*	0.333	0.100	1.000	0.167	0.125	0.167	0.100	0.000	0.444	0.000	1.000	0.100	0.375	1.000	1.000	1.000	–	0.250	0.167
**9**	0.107	0.250	0.111	0.067	*	1.000	1.000	0.333	1.000	1.000	1.000	1.000	0.250	0.250	0.333	0.250	0.444	–	1.000	1.000	1.000	0.250	0.048
**11**	0.111	0.100	–	0.004	0.184	*	0.048	0.429	0.400	1.000	1.000	0.048	1.000	0.048	1.000	0.048	0.333	–	1.000	1.000	1.000	0.313	1.000
**12**	0.007	1.000	1.000	1.000	0.143	0.002	*	0.267	0.333	1.000	1.000	0.200	1.000	0.250	1.000	0.286	0.083	1.000	1.000	0.250	1.000	1.000	0.476
**13**	0.010	0.000	0.063	0.028	0.048	0.086	0.022	*	1.000	0.133	1.000	0.444	0.083	0.389	0.167	0.333	0.222	1.000	1.000	1.000	1.000	0.458	0.222
**14**	0.004	0.063	0.250	0.008	0.333	0.100	0.067	0.640	*	1.000	1.000	1.000	0.333	0.400	0.200	0.400	0.100	–	–	1.000	1.000	1.000	0.438
**15**	0.090	0.100	0.063	0.010	0.250	0.022	0.028	0.003	0.086	*	0.333	1.000	0.214	0.286	0.357	0.083	0.278	1.000	1.000	1.000	–	0.500	0.278
**16**	0.033	0.100	0.167	0.004	0.259	0.038	0.107	0.212	0.143	0.067	*	1.000	1.000	1.000	1.000	1.000	1.000	1.000	1.000	1.000	1.000	0.250	0.188
**17**	0.048	0.063	–	0.000	0.200	0.002	0.022	0.127	0.556	0.036	0.063	*	0.200	1.000	1.000	1.000	1.000	–	1.000	1.000	–	1.000	1.000
**18**	0.002	0.250	0.375	0.127	0.025	0.167	0.583	0.005	0.067	0.018	0.167	0.022	*	0.400	1.000	0.444	0.083	1.000	1.000	1.000	1.000	1.000	0.214
**19**	0.007	0.063	1.000	0.000	0.036	0.002	0.036	0.068	0.100	0.048	0.048	0.143	0.100	*	1.000	1.000	1.000	–	1.000	0.286	1.000	1.000	0.267
**20**	0.048	0.063	1.000	0.125	0.111	0.082	0.238	0.010	0.022	0.128	0.036	0.086	0.333	1.000	*	1.000	1.000	–	1.000	0.357	1.000	1.000	0.000
**21**	0.012	0.063	1.000	0.004	0.036	0.002	0.048	0.037	0.100	0.007	0.083	0.143	0.127	1.000	1.000	*	1.000	1.000	1.000	0.286	1.000	1.000	0.200
**22**	0.063	0.063	1.000	0.063	0.127	0.037	0.002	0.028	0.010	0.020	0.114	0.286	0.005	0.656	0.583	0.667	*	1.000	1.000	0.167	1.000	0.458	0.125
**23**	0.030	–	0.063	0.074	–	–	0.038	0.091	–	0.450	0.450		0.057	–	–	0.038	0.065	*	1.000	–	–	1.000	1.000
**25**	0.267	–	0.063	0.100	0.086	0.028	0.016	0.083	–	0.022	0.028	0.429	0.048	0.086	0.067	0.063	0.120	0.012	*	1.000	1.000	1.000	1.000
**26**	0.048	0.063	0.333	0.143	0.082	0.107	0.036	0.250	0.357	0.036	0.036	0.250	0.357	0.048	0.128	0.048	0.010	–	0.048	*	1.000	1.000	0.167
**27**	0.259	0.375	1.000	–	0.020	0.048	0.250	0.111	0.143	–	0.016	–	0.238	0.438	1.000	0.438	0.167	–	0.020	0.438	*	1.000	1.000
**28**	0.133	0.063	0.063	0.025	0.036	0.058	0.107	0.080	0.143	0.167	0.025	0.063	0.184	0.107	0.082	0.141	0.080	0.267	0.038	0.036	0.016	*	0.350
**29**	0.063	0.500	0.375	0.008	0.002	0.238	0.149	0.028	0.123	0.020	0.010	0.286	0.046	0.022	0.000	0.010	0.016	0.046	0.057	0.010	0.111	0.034	*

Table S14. LD for males of Eldorado A ($n = 14$), where **2=** rs6639398, **5=** rs5986751, **7=** rs9781645,**8=** rs2209420, **9=** rs1299087, **11=**rs318173, **12=** rs933315, **13=** Yb8NBC102, **14=** Ya5DP62, **15=** MID3754, **16=** MID3756, **17=** Ya5NBC37, **18=** rs1991961, **19=** rs4825889, **20=** rs1781116, **21=** rs1937193, **22=** rs1781104, **23=** rs149910, **25=** MID1705, **26=** Yb8DP49, **27=** Ya5DP77, **28=** MID193, **29=** MID1540. Above diagonal: D'-values. Below diagonal: r^2-values.

	0	2	5	6	7	8	9	10	11	12	13	14	15	16	17	18	19	20	21	22	23	24	25	26	27	28	29
**0**	*	0.200	0.333	0.111	0.375	1.000	0.333	1.000	1.000	0.464	1.000	1.000	0.000	0.063	0.556	0.389	1.000	1.000	1.000	0.500	0.375	0.500	0.500	0.063	0.111	1.000	1.000
**2**	0.006	*	0.040	0.289	0.063	1.000	0.250	1.000	0.273	0.143	1.000	1.000	0.600	0.318	0.515	0.450	0.273	0.250	0.273	0.200	0.000	0.200	0.200	0.250	1.000	0.143	0.625
**5**	0.022	0.001	*	0.238	0.250	1.000	1.000	1.000	1.000	0.250	1.000	1.000	0.000	0.583	0.111	0.083	0.200	0.167	0.200	0.333	0.500	0.333	0.333	0.250	0.467	0.063	0.643
**6**	0.005	0.049	0.027	*	0.196	0.111	0.000	1.000	1.000	0.196	1.000	1.000	0.429	0.464	0.238	1.000	0.111	0.063	0.111	0.429	0.286	0.143	0.143	0.375	0.407	0.286	0.375
**7**	0.040	0.002	0.036	0.039	*	1.000	1.000	1.000	1.000	0.333	1.000	1.000	0.196	1.000	1.000	0.167	0.464	0.500	0.464	0.082	0.333	0.082	0.082	0.417	1.000	0.333	0.000
**8**	0.048	0.314	0.086	0.002	0.135	*	0.400	1.000	0.429	1.000	1.000	1.000	1.000	1.000	0.385	1.000	1.000	1.000	1.000	1.000	0.167	0.000	0.000	0.318	1.000	1.000	1.000
**9**	0.067	0.025	0.067	0.000	0.200	0.160	*	1.000	0.400	0.143	1.000	1.000	0.000	1.000	1.000	1.000	0.333	0.313	0.333	0.000	1.000	1.000	1.000	0.313	1.000	0.083	0.083
**10**	0.022	0.147	0.040	0.086	0.082	0.010	0.018	*	1.000	1.000	1.000	1.000	1.000	1.000	1.000	1.000	1.000	1.000	1.000	1.000	1.000	1.000	1.000	1.000	1.000	1.000	1.000
**11**	0.048	0.023	0.086	0.111	0.135	0.184	0.160	0.010	*	1.000	1.000	1.000	1.000	1.000	0.3846	0.083	0.333	0.318	0.333	0.000	1.000	0.000	0.000	0.318	1.000	1.000	1.000
**12**	0.069	0.009	0.036	0.039	0.083	0.135	0.006	0.082	0.135	*	1.000	1.000	0.388	0.333	0.286	0.500	0.464	0.417	0.464	0.082	0.300	0.196	0.196	0.417	0.375	0.000	0.143
**13**	0.022	0.030	0.111	0.086	0.082	0.010	0.018	0.004	0.010	0.082	*	1.000	1.000	1.000	1.000	1.000	1.000	1.000	1.000	1.000	1.000	1.000	1.000	–	1.000	1.000	1.000
**14**	0.026	0.026	0.107	0.107	0.077	0.005	0.022	0.005	0.011	0.077	1.000	*	1.000	1.000	1.000	1.000	1.000	1.000	1.000	1.000	1.000	1.000	1.000	–	1.000	1.000	1.000
**15**	0.000	0.164	0.000	0.143	0.039	0.143	0.000	0.067	0.143	0.115	0.067	0.063	*	1.000	0.333	1.000	0.000	0.063	0.000	0.250	0.063	0.250	0.250	0.063	0.333	0.082	0.388
**16**	0.001	0.101	0.186	0.069	0.273	0.026	0.022	0.026	0.026	0.030	0.026	0.031	0.416	*	0.318	0.500	1.000	1.000	1.000	0.063	0.300	0.464	0.464	1.000	0.546	0.417	0.125
**17**	0.214	0.135	0.002	0.010	0.135	0.092	1.000	0.015	0.092	0.018	0.015	0.018	0.026	0.043	*	0.083	0.111	0.091	0.111	0.333	0.444	0.333	0.333	0.091	0.180	0.286	0.286
**18**	0.068	0.096	0.005	0.476	0.008	0.083	0.063	0.083	0.002	0.167	0.083	0.083	0.476	0.167	0.002	*	1.000	1.000	1.000	0.267	0.010	0.267	0.267	0.000	0.267	0.200	0.200
**19**	0.111	0.055	0.022	0.005	0.069	0.429	0.067	0.022	0.048	0.069	0.200	0.286	0.000	0.091	0.009	0.185	*	1.000	1.000	1.000	0.375	0.500	0.500	0.091	1.000	0.531	0.063
**20**	0.132	0.046	0.015	0.002	0.100	0.423	0.058	0.026	0.043	0.052	0.196	0.282	0.002	0.109	0.006	0.250	1.000	*	1.000	1.000	0.300	0.464	0.464	0.067	1.000	0.563	0.000
**21**	0.111	0.055	0.022	0.005	0.069	0.429	0.067	0.022	0.048	0.069	0.200	0.286	0.000	0.091	0.009	0.185	1.000	1.000	*	1.000	0.375	0.500	0.500	0.091	1.000	0.531	0.063
**22**	0.083	0.018	0.067	0.143	0.005	0.143	0.000	0.067	0.000	0.005	0.067	0.082	0.063	0.002	0.026	0.071	0.333	0.318	0.333	*	0.286	0.000	0.000	0.531	1.000	0.388	0.196
**23**	0.034	0.000	0.083	0.048	0.083	0.006	0.267	0.107	0.231	0.067	0.048	0.058	0.003	0.020	0.074	0.010	0.034	0.020	0.034	0.048	*	0.688	0.688	0.222	0.167	0.417	0.000
**24**	0.083	0.018	0.067	0.016	0.005	0.000	0.143	0.067	0.000	0.039	0.067	0.082	0.063	0.069	0.026	0.071	0.083	0.069	0.083	0.000	0.360	*	1.000	0.464	0.333	0.196	0.388
**25**	0.083	0.018	0.067	0.016	0.005	0.000	0.143	0.067	0.000	0.039	0.067	0.082	0.063	0.069	0.026	0.071	0.083	0.069	0.083	0.000	0.360	1.000	*	0.464	0.333	0.196	0.388
**26**	0.001	0.011	0.011	0.034	0.052	0.043	0.058	0.026	0.043	0.052	–	–	0.002	0.109	0.006	0.000	0.006	0.003	0.006	0.117	0.010	0.069	0.069	*	1.000	0.125	0.000
**27**	0.009	0.508	0.084	0.049	0.176	0.033	0.040	0.289	0.033	0.040	0.015	0.018	0.026	0.205	0.032	0.022	0.077	0.091	0.077	0.231	0.005	0.026	0.026	0.091	*	1.000	0.375
**28**	0.219	0.009	0.003	0.048	0.083	0.176	0.002	0.063	0.176	0.000	0.082	0.103	0.005	0.052	0.018	0.040	0.117	0.169	0.117	0.115	0.174	0.039	0.039	0.008	0.219	*	0.082
**29**	0.219	0.223	0.241	0.107	0.000	0.176	0.002	0.082	0.176	0.020	0.063	0.058	0.115	0.008	0.018	0.040	0.002	0.000	0.002	0.039	0.000	0.115	0.115	0.000	0.040	0.005	*

Table S15. LD for males of Eldorado B ($n = 16$), where **0=** Ya5DP3, **2=** rs6639398, **5=** rs5986751, **6=** rs5964206, **7=** rs9781645,**8=** rs2209420, **9=** rs1299087, **10=** Yb8NBC634, **11=**rs318173, **12=** rs933315, **13=** Yb8NBC102, **14=** Ya5DP62, **15=** MID3754, **16=** MID3756, **17=** Ya5NBC37, **18=** rs1991961, **19=** rs4825889, **20=** rs1781116, **21=** rs1937193, **22=** rs1781104, **23=** rs149910, **24** rs652, **25=** MID1705, **26=** Yb8DP49, **27=** Ya5DP77, **28=** MID193, **29=** MID1540. Above diagonal: D'-values. Below diagonal: r^2-values.

	CORRIENTES				POSADAS				ELDORADOA				ELDORADOB			
Marker	PIC	PE	PD females	PD males	PIC	PE	PD females	PD males	PIC	PE	PD females	PD males	PIC	PE	PD females	PD males
**rs6639398**	0.222	0.047	0.412	0.255	0.233	0.051	0.429	0.269	0.322	0.116	0.563	0.403	0.311	0.105	0.547	0.385
**rs5986751**	0.360	0.164	0.609	0.471	0.375	0.187	0.625	0.500	0.375	0.188	0.625	0.500	0.363	0.167	0.612	0.476
**rs5964206**	0.348	0.146	0.595	0.449	0.341	0.137	0.586	0.435	0.057	0.003	0.111	0.058	0.374	0.186	0.624	0.498
**rs9781645**	0.363	0.167	0.612	0.476	0.365	0.171	0.614	0.480	0.360	0.164	0.609	0.471	0.341	0.137	0.586	0.435
**rs2209420**	0.341	0.137	0.586	0.435	0.384	0.186	0.634	0.499	0.351	0.151	0.599	0.455	0.233	0.051	0.429	0.269
**rs1299087**	0.373	0.185	0.623	0.497	0.363	0.167	0.612	0.476	0.363	0.167	0.612	0.476	0.367	0.174	0.617	0.484
**rs318173**	0.212	0.042	0.395	0.241	0.327	0.121	0.569	0.412	0.351	0.151	0.599	0.455	0.277	0.077	0.498	0.332
**rs933315**	0.370	0.179	0.620	0.490	0.375	0.187	0.625	0.500	0.252	0.062	0.460	0.295	0.374	0.186	0.624	0.498
**rs1991961**	0.351	0.151	0.599	0.455	0.367	0.174	0.617	0.484	0.375	0.188	0.625	0.500	0.374	0.186	0.624	0.498
**rs4825889**	0.363	0.167	0.612	0.476	0.332	0.127	0.575	0.420	0.355	0.155	0.603	0.461	0.336	0.132	0.581	0.428
**rs1781116**	0.369	0.177	0.618	0.487	0.332	0.127	0.575	0.420	0.351	0.151	0.599	0.455	0.351	0.151	0.599	0.455
**rs1937193**	0.369	0.177	0.618	0.487	0.336	0.132	0.581	0.428	0.344	0.142	0.591	0.442	0.344	0.142	0.591	0.442
**rs1781104**	0.373	0.185	0.623	0.497	0.367	0.174	0.617	0.484	0.365	0.171	0.614	0.480	0.374	0.186	0.624	0.498
**rs149910**	0.363	0.167	0.612	0.476	0.373	0.185	0.623	0.497	0.341	0.137	0.586	0.435	0.375	0.188	0.625	0.500
**rs652**	0.322	0.116	0.563	0.403	0.365	0.171	0.614	0.480	0.260	0.067	0.473	0.308	0.372	0.183	0.622	0.495

Table S16. Forensic parameters for SNPs. Corrientes (32 females, 60 males), Posadas (28 females, 24 males), Eldorado A (13 females, 14 males) and Eldorado B (11 females, 16 males). Markers with *PIC* values greater than 0.5 are considered very informative, with values between 0.25 and 0.50, informative and with values less than 0.25, not very informative.

	CORRIENTES				POSADAS				ELDORADOA				ELDORADOB			
Marker	PIC	PE	PD females	PD males	PIC	PE	PD females	PD males	PIC	PE	PD females	PD males	PIC	PE	PD females	PD males
**MID 3754**	0.371	0.181	0.621	0.493	0.365	0.171	0.614	0.480	0.277	0.077	0.498	0.332	0.375	0.188	0.625	0.500
**MID 3756**	0.375	0.187	0.625	0.499	0.341	0.137	0.586	0.435	0.341	0.137	0.586	0.435	0.371	0.181	0.621	0.493
**MID1705**	0.260	0.067	0.473	0.308	0.367	0.174	0.617	0.484	0.222	0.047	0.412	0.255	0.375	0.188	0.625	0.500
**MID193**	0.375	0.187	0.625	0.499	0.371	0.181	0.621	0.493	0.322	0.116	0.563	0.403	0.374	0.186	0.624	0.498
**MID1540**	0.365	0.171	0.614	0.480	0.370	0.179	0.620	0.490	0.375	0.187	0.625	0.499	0.374	0.186	0.624	0.498

Table S17. Forensic parameters for INDELs. Corrientes (32 females, 60 males), Posadas (28 females, 24 males), Eldorado A (13 females, 14 males) and Eldorado B (11 females, 16 males). Markers with *PIC* values greater than 0.5 are considered very informative, with values between 0.25 and 0.50, informative and with values less than 0.25, not very informative.

	CORRIENTES				POSADAS				ELDORADOA				ELDORADOB			
Marker	PIC	PE	PD females	PD males	PIC	PE	PD females	PD males	PIC	PE	PD females	PD males	PIC	PEPD females	PD males	
**Ya5DP3**	0.164	0.025	0.311	0.180	0.242	0.056	0.445	0.282	0.136	0.017	0.262	0.147	0.177	0.029	0.334	0.196
**Ya5DP4**	0.038	0.001	0.076	0.039	0.000	0.000	0.000	0.000	0.000	0.000	0.000	0.000	0.000	0.000	0.000	0.000
**Ya5 491**	0.020	0.000	0.039	0.020	0.020	0.000	0.039	0.020	0.000	0.000	0.000	0.000	0.000	0.000	0.000	0.000
**Ya5DP13**	0.000	0.000	0.000	0.000	0.000	0.000	0.000	0.000	0.000	0.000	0.000	0.000	0.000	0.000	0.000	0.000
**Yb8NBC634**	0.038	0.001	0.076	0.039	0.038	0.001	0.076	0.039	0.090	0.008	0.176	0.095	0.057	0.003	0.111	0.058
**Yb8NBC102**	0.327	0.121	0.569	0.412	0.305	0.099	0.539	0.375	0.327	0.121	0.569	0.412	0.136	0.017	0.262	0.147
**Ya5DP62**	0.332	0.127	0.575	0.420	0.305	0.099	0.539	0.375	0.327	0.121	0.569	0.412	0.136	0.017	0.262	0.147
**Ya5NBC37**	0.305	0.099	0.539	0.375	0.277	0.077	0.498	0.332	0.341	0.137	0.586	0.435	0.311	0.105	0.547	0.385
**Yb8DP49**	0.222	0.047	0.412	0.255	0.212	0.042	0.395	0.241	0.317	0.110	0.555	0.394	0.242	0.056	0.445	0.282
**Ya5DP77**	0.305	0.099	0.539	0.375	0.311	0.105	0.547	0.385	0.222	0.047	0.412	0.255	0.311	0.105	0.547	0.385

Table S18. Forensic parameters for Alus. Corrientes (32 females, 60 males), Posadas (28 females, 24 males), Eldorado A (13 females, 14 males) and Eldorado B (11 females, 16 males). Markers with *PIC* values greater than 0.5 are considered very informative, with values between 0.25 and 0.50, informative and with values less than 0.25, not very informative.
